# Time dependent effects of prolonged hyperglycemia in zebrafish brain and retina

**DOI:** 10.3389/fopht.2022.947571

**Published:** 2022-08-25

**Authors:** Cassie J. Rowe, Mikayla Delbridge-Perry, Nicole F. Bonan, Annastelle Cohen, Meg Bentley, Kathleen L. DeCicco-Skinner, Terry Davidson, Victoria P. Connaughton

**Affiliations:** ^1^ Department of Biology, American University, Washington, DC, United States; ^2^ Center for Neuroscience and Behavior, American University, Washington, DC, United States; ^3^ Department of Chemistry, American University, Washington, DC, United States; ^4^ Department of Neuroscience, and American University, Washington, DC, United States

**Keywords:** *Danio rerio*, diabetic complications, tight junctions, inflammation, neurotransmitters

## Abstract

Prolonged hyperglycemia causes long-term vision complications and an increased risk of cognitive deficits. High blood sugar also confers an osmotic load/stress to cells. We assessed behavioral and neurochemical changes in zebrafish brain and retina following prolonged hyperglycemia for 4-weeks or 8-weeks. At each time point, behavior was assessed using 3-chamber choice task and optomotor response; tissue was then collected and levels of inflammatory markers, tight junction proteins, and neurotransmitters determined using Western Blots. After 4-weeks, brain levels of v-rel reticuloendotheliosis viral oncogene homolog A (avian) (RelA; NF-kB subunit), IkB kinase (IKK), and glial fibrillary acidic protein (GFAP) were significantly elevated; differences in zonula occludens-1 (ZO-1), claudin-5, glutamic acid decarboxylase (GAD), and tyrosine hydroxylase (TH) were not significant. In retina, significant differences were observed only for TH (decreased), Rel A (increased), and GFAP (increased) levels. Glucose-specific differences in initial choice latency and discrimination ratios were also observed. After 8-weeks, RelA, GAD, and TH were significantly elevated in both tissues; IKK and GFAP levels were also elevated, though not significantly. ZO-1 and claudin-5 levels osmotically decreased in retina but displayed an increasing trend in glucose-treated brains. Differences in discrimination ratio were driven by osmotic load. OMRs increased in glucose-treated fish at both ages. *In vivo* analysis of retinal vasculature suggested thicker vessels after 4-weeks, but thinner vessels at 8-weeks. *In vitro*, glucose treatment reduced formation of nodes and meshes in 3B-11 endothelial cells, suggesting a reduced ability to form a vascular network. Overall, hyperglycemia triggered a strong inflammatory response causing initial trending changes in tight junction and neuronal markers. Most differences after 4-weeks of exposure were observed in glucose-treated fish suggesting effects on glucose metabolism independent of osmotic load. After 8-weeks, the inflammatory response remained and glucose-specific effects on neurotransmitter markers were observed. Osmotic differences impacted cognitive behavior and retinal protein levels; protein levels in brain displayed glucose-driven changes. Thus, we not only observed differential sensitivities of retina and brain to glucose-insult, but also different cellular responses, suggesting hyperglycemia causes complex effects at the cellular level and/or that zebrafish are able to compensate for the continued high blood glucose levels.

## 1 Introduction

Prolonged hyperglycemia associated with diabetes eventually leads to visual complications (1) and an increased risk of cognitive deficits (2). Hyperglycemia-induced changes in retinal vasculature (3) are used to clinically diagnose diabetic retinopathy and are believed to subsequently compromise the neural retina resulting in vision loss (4). Mechanisms underlying cognitive impairments associated with prolonged hyperglycemia are similar to those in retina due to structural and functional similarities between the blood-brain-barrier (BBB) and blood-retinal-barrier (BRB). Given these similar mechanisms, it has been suggested that changes in retinal vasculature may be indicative of changes in brain vasculature and/or memory impairment (5–7). A direct comparison between hyperglycemia-induced retinal changes and brain changes was performed in a Type 2 diabetes rodent model (8). In this study, BRB and BBB permeability was assessed 16 weeks (4 months) after inducing Type 2 diabetes with a high fat diet followed by streptozotocin injection. At that time, increased BRB permeability, assessed using albumin-bound dye, was noted; BBB permeability was also increased, but to a lesser extent that was not significant. However, both tissues displayed histological damage and increased protein levels of the proinflammatory cytokine ICAM-1 (8). These results suggest similar pathology, but different time course, to hyperglycemic insult in retina and brain.

Prolonged/uncontrolled hyperglycemia initiates oxidative stress, the formation of reactive oxygen species (ROS), and an inflammatory response (9) (reviewed in (10)). This oxidative stress impairs both glucose uptake to cells and insulin secretion, maintaining high blood sugar levels (10). Increased levels and/or expression of pro-inflammatory cytokines (TNFα, IL-6) (11) activate NF-κB and its downstream pathways (12, 13) causing disruption of the BBB (11, 12, 14). At the BBB, vascular endothelial cells produce ROS (10, 14) and compromised glial cells trigger further inflammation (12). Permeability increases as levels of tight junction proteins (claudin-5, occludin, ZO-1) are decreased (14, 15). In animal models, this BBB breakdown is associated with memory/cognitive impairment (15–17), identifying a link between BBB breakdown, inflammation, and cognitive deficits.

Our lab (18–20) and others (21–24) use the zebrafish model to study diabetic complications. Protocols for inducing hyperglycemia, either non-invasively (18–20, 25) or following streptozotocin injection (23, 24) have been described, and subsequent changes in retinal morphology have been reported. After 1-month of hyperglycemia, zebrafish retinas are thinner (18, 23, 24), basement membranes of retinal vessels are thicker and endothelial cell junctions are altered (21). There is also a loss of cone photoreceptors (21) and decreased b-wave amplitudes in glucose-treated fish compared to mannitol-treated controls (20). Neurochemically, 4-weeks of exposure increases retinal glial fibrillary acidic protein (GFAP) and nuclear factor k-light-chain-enhancer of activated B cells (NF-κB) levels, indicating an inflammatory response (20). In other animal models, changes in γ-aminobutyric acid (GABA) (26–30) and dopamine (31, 32) systems are also reported in hyperglycemic retinal tissue; it is not known if these systems are altered in the zebrafish model. Neurochemical changes in these markers in hyperglycemic brains are not known; however, reduced brain (hippocampal) size and white matter atrophy are reported (33), suggesting compromised neurons.

The overarching goals of this study were to determine if (1) there are parallel neurochemical changes in retinal and brain tissue in response to hyperglycemic insult and (2) these changes correlate with behavioral deficits. We hypothesized that prolonged hyperglycemia would lead to cognitive and visual function impairment in adult zebrafish, with the degree of impairment increasing with duration of exposure. We also hypothesized that behavioral changes would be correlated with neurochemical changes in brain and retina.

## 2 Methods

### 2.1 Animals

Adult wild-type zebrafish (*Danio rerio*) aged 6-12 months (N=96) were obtained as embryos from a commercial supplier (Live Aquaria, Rhinelander, WI, USA) and reared in-house at the Zebrafish Ecotoxicology, Neuropharmacology, and Vision (ZENV) laboratory at American University. Fish were maintained in an Aquatic Habitats (AHAB; Pentair Aquatic Ecosystems, Apopka, Fl, USA) rack system, at 28-29°C on a 14 hours (hr) light: 10 hr dark photoperiod. Fish were fed twice per day with commercial flakes (TetraMin™, Blacksburg, VA, USA) and enriched with either dry brine shrimp (Omega One LLC, Aurora, IL, USA) or live *Artemia* (Connecticut Valley Biological, Southhampton, MA, USA). All experimental procedures were approved by the Institutional Animal Care and Use Committee (IACUC) at American University (protocol #1606, #1902).

### 2.2 Induction of hyperglycemia

To induce hyperglycemia, adult zebrafish were transferred to 4 L experimental tanks. The tanks were placed in temperature-controlled water baths at 28-29°C and aerated. Temperature, pH, and other environmental parameters were recorded daily and were within normal limits of the stock holding tanks as in [19]. Fish were fed a dry mixture (TetraMin™ flakes, dried brine shrimp) daily while in the experimental containers and prior to transfers.

**Figure 1:**
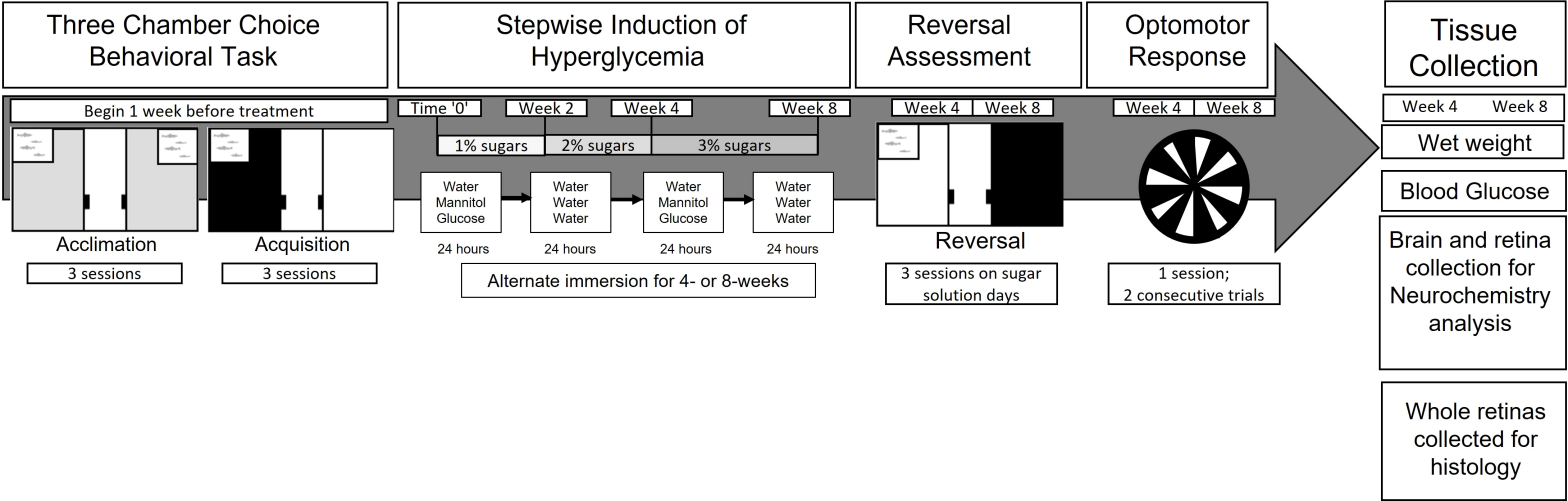
Experimental Design. Prior to induction of hyperglycemia, naïve zebrafish underwent six days of acclimation and acquisition behavioral training. Then, for a period of 4- or 8-weeks, zebrafish were treated with either mannitol or glucose in a stepwise manner. After treatment, behavioral assessment (three-chamber choice; OMR) was performed, followed by euthanasia, wet weight and blood glucose measurements, and tissue collection.

Hyperglycemia in wild-type zebrafish was induced using a stepwise alternate immersion in D-glucose (MilliporeSigma, St. Louis, MO, USA) with a treatment regime (19, 25) duration of 4-weeks (n=48) or 8-weeks (n=48; **Figure 1**). In brief, adult zebrafish (n=32) were exposed to a 1% glucose solution for 2-weeks, followed by a 2% solution for 2-weeks, and then 3% glucose for 4-weeks. Exposure to sugar alternated, with fish exposed to glucose for 24 hr, after which they were exposed to water for 24 hrs. Two control treatments were used: water-treated controls (n=32) alternated between 0% glucose/0% glucose (water) solutions every 24hrs (handling control) and D-mannitol (Acros Organics, Fair Lawn, NJ, USA)-treated controls (n=32) alternated between 1-3% mannitol/0% mannitol every 24 hr (osmotic control) with the concentration of mannitol the same as the glucose concentration. All treatment groups included both male and female fish.

Separate one-way ANOVAs were used to assess changes in wet weight and blood glucose levels after 4- and 8-weeks of treatment. Significance was evaluated at α = 0.05.

### 2.3 Three-chamber choice task

The three-chamber choice task in our zebrafish model has been described and videoed previously (34). The behavioral chamber was a 40 L aquarium (50×30×30 cm^3^; Petsmart, Phoenix, AZ, USA) modified to have a central chamber (10×30×30 cm^3^), separated from two side choice chambers (each 20×30×30 cm^3^), as in (34, 35). During experiments, approximately 30 L of Deer Park Brand ‘control’ water (Nestlé Waters North America, Stamford, CT, USA) was added to the tank. Two submersible aquarium heaters (Marineland, Los Angeles, CA, USA) were added for 24 hr prior to testing to bring the temperature to 28.5°C; the heaters were removed at the start of the behavior session. A full water change typically occurred after two days of use (34). For the discrimination task, colored felt pieces (beige, black, or white) were individually placed on the outer back, side, and bottom of the side choice chambers using Adhesive Sticky Back Hook and Loop Mounting Squares (Velcro, Manchester, NH, USA). The overall experimental design for these tests involved three phases - acclimation, acquisition, and reversal - with hyperglycemic induction occurring between acquisition and reversal (34).

Acclimation to the behavioral chamber occurred over three days: two days of group acclimation followed by one day of individual acclimation. Briefly, for group acclimation, six zebrafish were placed into the center chamber and allowed to roam freely for 30 min. In both choice compartments, a beige (neutral) background was used, and a live shoal was present. The shoal was created by placing four adult zebrafish not otherwise used in the study in a small, clear tank in the far back corner of each choice chamber. The shoal fish were chosen randomly from stock tanks each day and included at least one male and one female that were age- and sized-matched to the experimental fish. A fish was considered to have entered one of the side chambers when its entire body entered the chamber. During individual acclimation, each zebrafish was placed in the center starting chamber for 2 min with the sliding doors closed. Then, both doors were opened simultaneously, and the fish was rewarded (i.e., able to interact with the shoal) for swimming entirely through either door. Each fish was required to swim from the central chamber through a door a total of 10 times, regardless of which side. If a fish was unable to complete this task, it was excluded from the study.

After acclimation, zebrafish began a 3-day acquisition phase. During this phase, the background of one of the side chambers was covered in white felt and the background of the other chamber was covered in black felt. Background color of each side alternated using a pseudorandom schedule (36). As above, single fish were placed in the starting chamber for 2 min before both doors were simultaneously opened. Using a biased design, fish were randomly assigned either a black (B+/W-) or white (W+/B-) preference. If the fish correctly chose their preferred color, the door to the center chamber was immediately closed and the fish was restricted to the preferred side for one minute. This trial was scored as “C” for “Correct” (i.e., the fish chose the side of the tank with the shoal reward). If the fish swam through the incorrect door, they were transferred back into the center chamber, both doors were closed, and this trial was scored as “I” for “Incorrect” (the fish did not choose the side with the shoal reward). If the fish did not decide within 2 min after the doors opened, the fish was moved to the correct side and denoted “M” for “Marked” (or force-rewarded, meaning the fish was placed on the side with the shoal reward). Between each trial was a 1 min period in the center chamber. Each fish underwent eight trials during each training session.

For each individual fish, initial choice latency (time to first decision) was recorded in addition to their side preferences during each trial. Results are reported as group averages for each acquisition day. Based on their performance, fish were also sorted into ‘high performing’ and ‘low performing’ groups. A fish was considered high performing if it successfully chose the correct side of the tank in at least 6 of the 8 total trials for the day. Any fish who did not meet this criterion was considered a low performer.

Following the acquisition phase, trained zebrafish remained in their performance group for the duration of the experiment and hyperglycemic induction. After 4- or 8-weeks of hyperglycemia, zebrafish underwent a 3-day reversal assessment where the rewarded side was reversed: fish previously rewarded on the white side were now rewarded on the black side (and vice versa). For each fish, initial choice latency and the number of force-rewarded trials were recorded for each individual fish. Results were reported as group averages of eight trials on each reversal day. The data was analyzed in two ways. First, a two-way ANOVA (α = 0.05) identified significant differences in the entire experimental population (treatment and reversal session as the main variables). However, the subsequent Tukey-Kramer *post-hoc* tests did not differentiate among treatment groups at each of the behavior sessions. Therefore, to determine treatment-specific changes a one-way ANOVA was conducted for each of the three reversal days.

Discrimination ratios for each fish were calculated daily following reversal behavior sessions and reported as the number of correct trials over the number of total trials $\;\left(\frac{\#C}{8\;trials}\right)$ . We also analyzed discrimination ratios during reversal using a two-way ANOVA to identify differences across the entire experimental population (treatment and trial days as the main variables). However, we were unable to differentiate treatment-specific differences using subsequent *post-hoc* multiple comparison at each behavioral session. Therefore, one-way ANOVAs with training session as the main variable were used to reveal treatment-specific effects on each trial day followed by Tukey-Kramer *post-hoc* tests to determine the source of significance.

### 2.4 Optomotor response (OMR)

Adults were removed from treatment tanks and placed individually in a 12-inch glass bowl placed on top of a 36-inch computer monitor (37, 38). A 12-inch diameter rotating black and white radial grating stimulus was projected onto the monitor and rotated (clockwise and counterclockwise) with a control, gray screen in between. Each stimulus was shown to the fish for 30 sec. The grey screen acted as a within-replicate control and allowed the fish to rest if needed. The sequence was run twice to ensure behavioral consistency. The responses to the entire OMR sequence were recorded on a VIXIA HFR700 HD video camera with 32x optical zoom, and 57x advanced zoom (Canon; Ota City, Tokyo, Japan). Recordings were made by one investigator and later analyzed by another trained, blinded observer. Each 30 sec interval was scored by tallying the number of total revolutions made by each fish and averaged for the two complete trials.

A performance ratio for each individual fish was calculated based on the total number of revolutions each fish made during two trials when the stimulus was on divided by the total number of revolutions for two trials when the stimulus was off $\left(\frac{\#\;of\;Revolutions\;\left(Stimulus\;On\right)}{\#\;of\;Revolutions\;\left(Stimulus\;Off\right)}\right)$ . Untreated fish (n=10) removed from stock tanks (AHAB group) and immediately tested were used as a control for the daily handling/transferring of fish (20). Our data revealed differences in OMR between fish directly removed from stock tanks and treated fish, suggesting a handling stress. Consequently, we normalized the performance ratio of each treatment group to the in-study water treated group at the same time point. This water-normalized performance ratio for mannitol and glucose at both timepoints is only for the presentation of graphical data. A one-way ANOVA was used to assess the data at each experimental endpoint for significant differences with α=0.05.

### 2.5 Neurochemistry

Following behavioral assessment, a subset of animals (n=16 per treatment/time point) were anesthetized in a 0.02% tricaine solution, weighed (to obtain wet weight; Sartorius balance), and decapitated. Blood glucose was measured from cardiac blood using a FreeStyle Lite Blood Glucose Meter (19, 39). Whole brains and retinas were collected and placed into NP-40 buffer (5% 1M Tris, pH 8.0, 0.9% NaCl, 1% Triton X-100) containing 0.1% protease inhibitors (MilliporeSigma, St. Louis, MO, USA) and stored at -80°C for protein isolation and Western Blots.

Retinal tissue was pooled from two animals to obtain enough protein for analysis, while individual whole brains could be used. A total of 3-5 replicates/treatment/time point for both tissue types were used throughout the neurochemistry analysis. Protein concentrations were determined using the Pierce BCA Protein Assay Kit (Thermo Fisher Scientific, Waltham, MA, USA). To prepare for gel electrophoresis, 20 μg of each sample, in addition to appropriate amounts of both NP-40 and 5x loading dye, were combined, and heated at 70°C for 10 min. The gel electrophoresis ran on NuPAGE 4-12% Bis-Tris gels (Invitrogen, Carlsbad, CA, USA) using 1x NuPAGE MOPS SDS Running Buffer (Invitrogen, Carlsbad, CA, USA). Pageruler plus prestained protein ladder (Thermo Fisher Scientific, Waltham, MA, USA) determined protein sizes. The gel electrophoresed for 80 min at 125 V, after which the proteins from the gel were transferred to a membrane in the Power Blotter Select Transfer stack (Invitrogen, Carlsbad, CA, USA), with the iBlot2 Gel Transfer Device (Invitrogen, Carlsbad, CA, USA) using the standard P0 setting for a total run time of 7 min. Following a successful transfer, membranes were blocked in 5% milk in 1X Tris-buffered Saline – Tween 20 (TBST; VWR, Radnor, PA, USA) at 4°C overnight on a shaker.

We used eight different primary antibodies and two secondary antibodies (**Supplemental Table 1**). The primary antibody in 5% milk was applied to the membranes on the shaker overnight. The next day, membranes were washed 3x in TBST for 5 min. The corresponding secondary antibody in 5% milk was applied to the membranes on the shaker for 45 min. The membranes were again washed 3x with TBST for 10 min. The chemiluminescent SuperSignal West Dura Extended Duration Substrate (Thermo Fisher Scientific, Waltham, MA, USA) was applied to the membranes for 5 min and visualized using a UVP Imaging System (Analytik Jena AG, Jena, Germany). After imaging, the membranes were stripped using Restore Western Blot Stripping buffer (Thermo Fisher Scientific, Waltham, MA, USA) and then blocked on 5% milk for at least 1 hr before probing with another antibody. No more than 3 antibodies were used on each membrane. Densitometry was performed on the Western Blot images using NIH Image J software. All results were normalized to the β-actin band. Differences in mean protein levels were assessed using a one-way ANOVA (α = 0.05).

### 2.6 Immunohistochemistry of retinal flatmounts

Whole eyes were placed into a petri dish containing Ames’ Medium (MilliporeSigma). Retinas were dissected from adult eyes and fixed for 15 min at room temperature with 4% Paraformaldehyde and then rinsed 3X, 20 min each, in phosphate buffered saline (PBS). Flatmounts were transferred to a 24-well dish for staining and blocked overnight using 10% normal goat serum (NGS) and 0.5% triton in PBS. ZO-1 primary antibody was added to each culture well and was left on for 5 days at 4°C. The primary antibody (**Supplemental Table 1**) was removed and flatmounts were washed 3x, 20 mi in well, in 1X PBS and secondary antibody was applied for an additional two days. An Olympus compound microscope (BX61) was used to image retinal vasculature at 20x magnification. The number of vessels and vessel thickness were measured using the measure tool on NIH ImageJ at a radius of 150 μm from the optic nerve.

### 2.7 In vitro tube formation assay

3B-11 cells were cultured in sub-confluent conditions in supplemented DMEM containing either glucose or mannitol (25 mM) until they reached passage 6 (as in (40)). The day before the assay, 3B-11 cells were serum starved by aspirating the media and replacing the supplemented DMEM with reduced supplemented DMEM (0.2% FBS, 2 mM L-glutamine, 1 mM sodium pyruvate, 100 U/mL penicillin, and 100 μg/mL streptomycin). Cells were grown for an additional 24 hours.

On the day of the assay, fresh media containing glucose and mannitol were made. Calcein-AM was added to the endothelial cells into the media at a final concentration of 2 μg/mL. 3B-11 cells were then washed with DPBS to remove excess stain. Cells were trypsinized by adding 1 mL trypsin-EDTA to each of the T-75 flasks containing cells for the assay. After trypsinization was completed, trypsin was neutralized by resuspending cells to a final volume of 10 mL in 10 DMEM, 10% FBS, 2 mM L-glutamine, 1 mL sodium pyruvate, 100 U/mL penicillin, and 100 μg/mL streptomycin. Cells were then filtered through 100 μm cell strainers to remove clumps. Cells were then counted and resuspended to a final concentration of 7.5 x 106 cells/mL in DMEM, 10% FBS, 2 mM L-glutamine, 1 mL sodium pyruvate, 100 u/mL penicillin, and 100 μg/mL streptomycin. To prep the culture plates, BME was added to each well of a 24-well dish and incubated at 37°C and 5% CO2 for 30 minutes in order to solidify the BME. Once the BME was set, 300 μL of conditioned media (Control, Mannitol, and Glucose) was added to the specific culture wells for each condition with 10 μL of the resuspended 3B-11 cells (approximately 75,000 cells). Tubes began to form two hours into the assay and were imaged every hour for 12 hours, though peak tube formation occurred after 6 hours.

Images were then processed so they fit the requirements of the angiogenesis analyzer plugin for ImageJ (NIH). Briefly, images were converted to an 8-bit image, contrast was enhanced and normalized (0.1%), images were then smoothed and despeckled. Tubes were quantified using the angiogenesis analyzer. The number of tubes, meshes, and nodes were calculated per 400 x 400 dpi image. Each image was run through the plugin twice and the quantifications were averaged. Each treatment had at least two images from two separate tube formation assays.

### 2.8 Statistical Analyses

All analyses described above were conducted using SPSS 25 (IBM) software package for Mac and graphs were constructed using Graphpad Prism 9. For all one-way or two-way ANOVAs, the Tukey-Kramer test for pairwise comparisons was used to assess the source of significance. All assumptions for normality, equal variance, and sample independence were met. Data are presented as mean ± standard error of the mean (SEM) unless otherwise noted.

## 3 Results

### 3.1 Changes after one month (4-weeks) of hyperglycemia

#### 3.1.1 Wet weight and blood glucose

**Figure 2:**
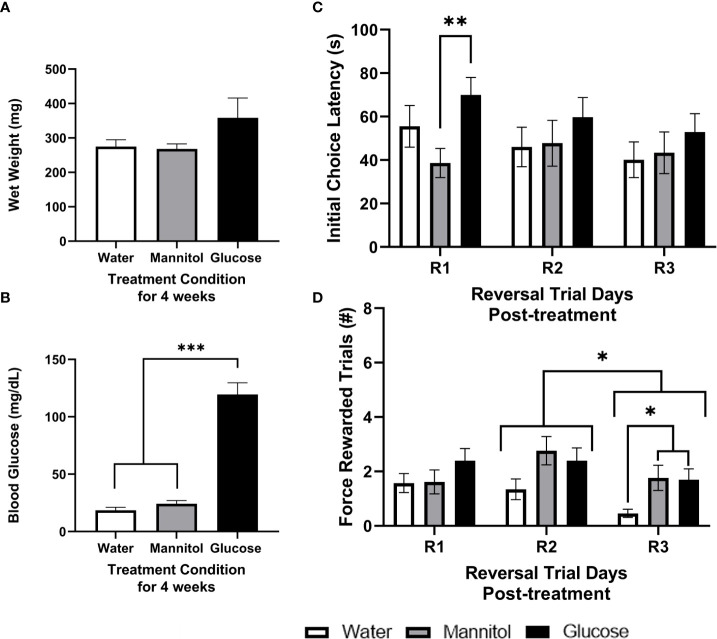
Health assessment, initial choice latency, and force-rewarded trials after 4-weeks of glucose treatment. Wet weight **(A)** and blood glucose **(B)** measurements taken from fish exposed to either alternating glucose, alternating mannitol, or alternating water conditions for 4-weeks. No significant differences in **(A)** wet weight were found across treatment groups (timepoint: *p*=0.146; treatment: *p*=0.067; timepoint x treatment *p*=0.478). However, **(B)** glucose-treated zebrafish exhibited significantly higher blood glucose levels (p<0.001). During reversal **(C)** there was a main effect of treatment on initial choice latency (R1, R2, R3; p=0.037). Further analyses revealed a significant difference between mannitol and glucose treatment groups on R1 (p=0.015). Significant main effects of training day (p=0.038) and treatment (p=0.004) were observed in the number of force-rewarded trials during reversal **(D)**. Specific differences between treatment groups occurred on R3 when the water-treated group was significantly reduced compared to mannitol- (p=0.039) and glucose-treated (p=0.042) fish. Data is presented as mean ± SE. Asterisks denoting statistical significance.

Adult zebrafish alternately immersed in glucose for 4-weeks displayed increased blood glucose levels compared to controls, though no significant difference in weight was observed. Mean wet weight in water or mannitol treated zebrafish averaged 271.5 ± 17.28 mg, while the wet weight of glucose treated fish averaged 358.5 ± 57.62 mg (**Figure 2A** **;** p=0.160). Mean blood glucose concentrations of glucose-treated fish were significantly increased averaging 119.5 ± 10.25 mg/dL compared to controls that averaged 21.4 ± 2.75 mg/dL (p<0.0001; **Figure 2B**).

#### 3.1.2 Initial choice latency *and* force rewarded trials

During reversal, treatment significantly impacted initial choice latency (p=0.037) when the responses of all fish were combined, with no other effects or interactions noted (training day: p=0.438; training day x treatment: p=0.693; **Figure 2C**). This effect was likely driven by specific differences on reversal day 1 (R1), when glucose-treated fish took longer to reach a first decision than mannitol-treated fish (p = 0.008). We also identified a main effect of both treatment and day (day: p=0.038; treatment: p=0.004) on the number of force-rewarded trials, with day 2 (R2) significantly different from day 3 (R3) (p=0.033; **Figure 2D**). Both glucose- and mannitol-treated fish were significantly different from the water group (water vs. mannitol: p=0.022; water vs. glucose: p=0.006), though there was no difference between these two groups (p=0.935). Analysis of differences across treatment groups and day revealed a significant difference only during R3 (p=0.021), when the water treatment group was significantly different from both mannitol (p=0.039) and glucose (p=0.042) treatment groups.

After 4-weeks of treatment, fish initially classified as high performing displayed no differences in initial choice latency (day: p=0.753; treatment: p=0.889; day x treatment p=0.564) and no difference in average force-rewarded trials (day: p=0.053; treatment: p=0.981; day x treatment: p=0.201; **Supplemental Figure 1A**). However, initial choice latency of low performing fish was significantly affected by treatment (p<0.001). This difference was observed on R1, when a significantly longer (p = 0.002) choice latency was observed in glucose-treated fish vs. both water- and mannitol-treated controls (**Supplemental Figure 1B**). Low performing fish also displayed significantly reduced responses in the water-treated group on all reversal days (p<0.001). The difference on R1 occurred between water vs glucose treatment groups (p=0.017); while on R3 the difference was between the water vs mannitol treatment groups (p=0.025). On R2, there was a main effect of treatment (p=0.045), though *post-hoc* analysis did not identify a specific difference on R2.

#### 3.1.3 Discrimination ratio

**Figure 3:**
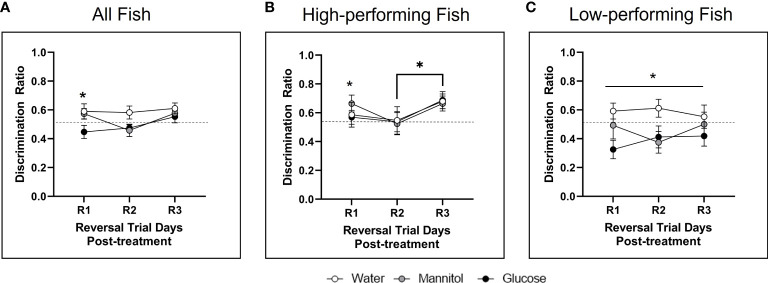
Discrimination ratio following 4-weeks of hyperglycemia. During reversal, analysis of **(A)** all fish identified a significant effect of treatment (p=0.048) with glucose-treated animals significantly different from water-treated controls on R1 (p=0.037). The high performing fish **(B)** displayed a main effect of day (p=0.041), with ratios during R2 different from R3. Differences across treatment were also noted on R1. For low performing fish **(C)**, there was a main effect of treatment (p=0.004). Asterisks denote statistical significance.

During reversal, analyzing the responses of all fish identified a significant main effect of treatment on discrimination ratio (p=0.048; **Figure 3A**). *Post-hoc* analyses revealed a main effect of treatment on R1 due to a significant difference between the glucose and mannitol treatment groups. High performing fish displayed a main effect of trial day (p=0.041) on the reversal discrimination ratio with a significant difference between R2 and R3 (**Figure 3B**). *Post-hoc* analyses identified a separate main effect of treatment (p=0.021) on R1, due to differences between the mannitol vs water and the mannitol vs glucose treatment groups. Low performing fish displayed a main effect of treatment after 4-weeks of exposure (p=0.004; **Figure 3C**) with a significant difference between the water vs glucose treatment groups (p=0.003). No difference was identified between the water- and mannitol-treated groups.

#### 3.1.4 Optomotor responses

Glucose-treated fish exhibited a stronger OMR performance ratio compared to controls. Surprisingly, the 2.25x increase observed in glucose-treated fish was not significant (p=0.570; **Supplemental Figure 2A**).

#### 3.1.5 Neurochemistry

**Figure 4:**
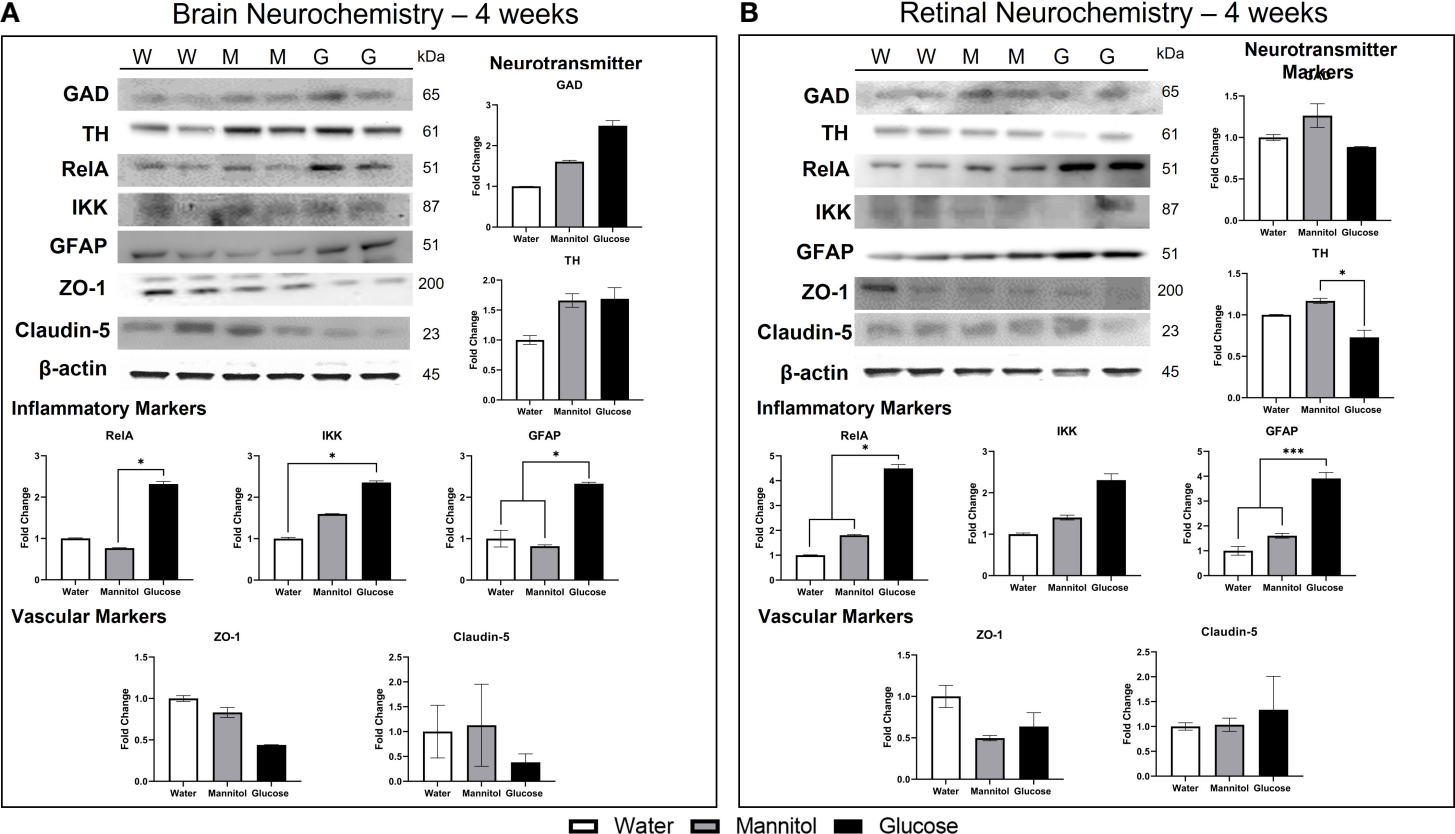
Brain and retinal neurochemistry after 4-weeks of treatment. **(A)** Brain neurochemistry changes after 4-weeks of treatment. Neurotransmitter markers: GAD levels increased 1.5-2x in glucose-treated tissue. The increase in TH levels was osmotic. Inflammatory markers: RelA (NF-κB), IKK, and GFAP levels were all significantly increased in glucose-treated tissue. Vascular markers: Tight junction proteins ZO-1 and claudin-5 were decreased in glucose-treated tissue, though not significantly. **(B)** Retinal neurochemistry after 4-weeks of hyperglycemia. Neurotransmitter markers: Levels of TH and GAD in retinal tissue were differentially affected by glucose exposure. GAD levels were unchanged, while TH levels decreased 0.2x, a significant finding. Inflammatory markers: Glucose treatment increased all inflammatory markers: RelA (4.5x), IKK (1-2.5x), and GFAP (4x) in retinal tissue. Vascular markers: ZO-1 levels decreased, while claudin-5 levels were increased. Representative Western Blots are shown at the top left of both panels **(A)** and **(B)**, with W, water treated; M, mannitol treated; G, glucose treated. Each sample was run in technical replicates and protein levels were normalized to β-actin. Data in all graphs are mean ± SE. Asterisks denote statistical significance. White bars, water-treated; gray bars, mannitol-treated; Black bars, glucose-treated.

In brain homogenates (**Figure 4A**), TH protein levels were not different across all treatment groups (p=0.105), though higher values were found in glucose- and mannitol-treated tissue. There was a 2x increase in GAD protein levels in glucose-treated tissue, though these differences were also not significant (p=0.065). In contrast, all inflammatory markers (RelA, p=0.031; IKK, p=0.029; and GFAP, p=0.012) were significantly upregulated in glucose-treated tissue, suggesting inflammation and reactive gliosis. Levels of the tight junction proteins ZO-1 (p=0.123) and claudin-5 were lowest in glucose-treated brain samples; however, these differences were not significant (p= 0.469).

In retinal homogenates (**Figure 4B**), there was no change in GAD protein levels (p=0.549), though levels in glucose-treated tissue were reduced compared to levels in mannitol-treated tissues. In contrast, a significant decrease in TH in glucose-treated tissue (p=0.05) was observed. All inflammatory markers were upregulated in response to glucose, with a 4x increase in RelA protein levels (p=0.025), a non-significant 2x increase in IKK (p=0.647), and a 4x increase in GFAP (p<0.001). ZO-1 protein levels in glucose- and mannitol-treated tissue were lower than values from water-treated tissue, whereas claudin-5 levels were highest in glucose-treated retinas. However, these differences in tight junction proteins were not significant (ZO-1 p=0.511; claudin-5 p=0.654).

### 3.2 Changes after two months (8-weeks) of hyperglycemia

#### 3.2.1 Wet weight and blood glucose

**Figure 5:**
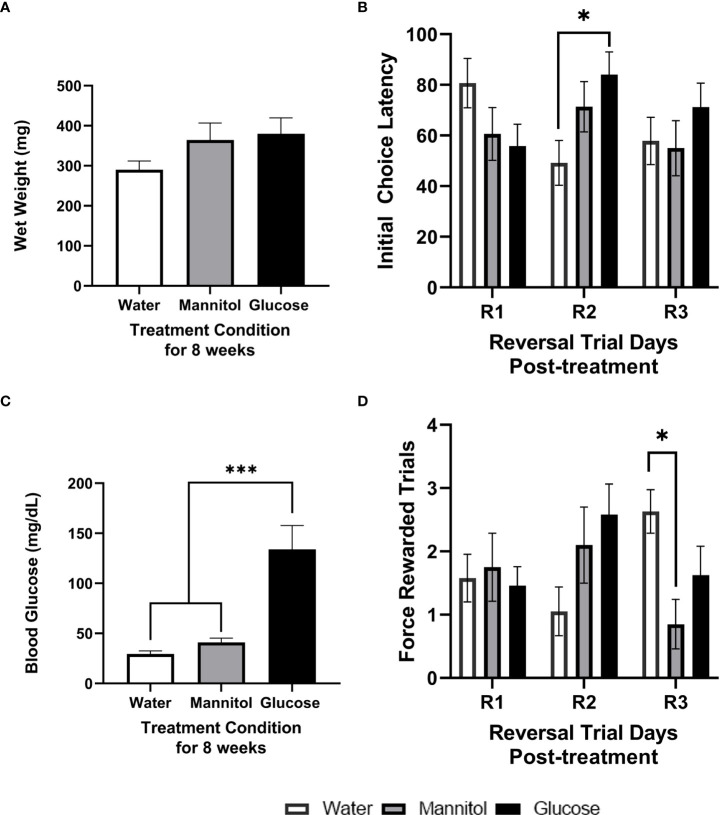
Health assessment, initial choice latency, and force rewarded trials after 8-weeks of glucose treatment. Wet weight **(A)** and blood glucose **(B)** measurements taken from fish exposed to either alternating glucose, alternating mannitol, or alternating water conditions for 8-weeks. No significant differences in **(A)** wet weight were found across treatment groups (timepoint: *p*=0.146; treatment: *p*=0.067; timepoint x treatment *p*=0.478). However, **(B)** glucose-treated zebrafish exhibited significantly higher blood glucose levels compared to both controls (p<0.001). During reversal, there was a significant interaction of training day and treatment on initial choice latency **(C)**, with a significant difference between water- and glucose-treated groups on R2 (p=0.010). The number of force-rewarded trials during reversal **(D)** displayed a significant day x treatment interaction (p=0.013), and a difference between water- and mannitol-treated groups on R3 (p=0.017). For all graphs, bars represent mean values ± SE. Asterisks denote statistical significance.

There was no difference in wet weight after 8-weeks of exposure (**Figure 5A**; p=0.213). Mean wet weight in water- or mannitol-treated groups ranged from 268.79 to 406.9 mg, while the wet weight of glucose treated fish averaged 379.9 ± 39.84 mg. As expected, mean blood sugar levels of glucose treated fish were significantly increased in glucose treated fish, with controls averaging 35.18 ± 3.72 mg/dL and glucose-treated fish averaging 133.9 ± 23.79 (p<0.001; **Figure 5B**).

#### 3.2.2 Initial choice latency and force-rewarded trials

A significant interaction between treatment and day was detected (p=0.034) across the three days of reversal training (**Figure 5C**), with a significant difference between water vs glucose treatment groups observed on R2 (p=0.010). Differences in the number of force-rewarded trials during reversal identified a significant treatment*day interaction (p=0.013), with a significant difference between mannitol vs water treatment groups (p=0.013) on R3 (**Figure 5D**).

After 8-weeks of treatment, high performers (**Supplemental Figure 3A**) displayed no significant main effects (day: p=0.860; treatment: p=0.212) or interactions (p=0.584). However, there were two significant and separate main effects of treatment (p=0.036) and day (p=0.041), but no interaction (p=0.250) on the number of force-rewarded trials. On R2, there were significantly more marked fish in the glucose-treatment group than in the water-treated controls (p=0.049). A similar trend was observed on R3. No significant differences due to treatment were observed on R1 (p=0.966).

The initial choice latency of low performers (**Supplemental Figure 3B**) showed a significant interaction between day and treatment (p=0.039) though statistical analysis revealed no significant main effect of treatment on any reversal day (p>0.05 for all). However, there was no significant differences in force-rewarded trials due to treatment (p=0.332) or day (p=0.387) for the low performing fish.

#### 3.2.3 Discrimination ratio

**Figure 6:**
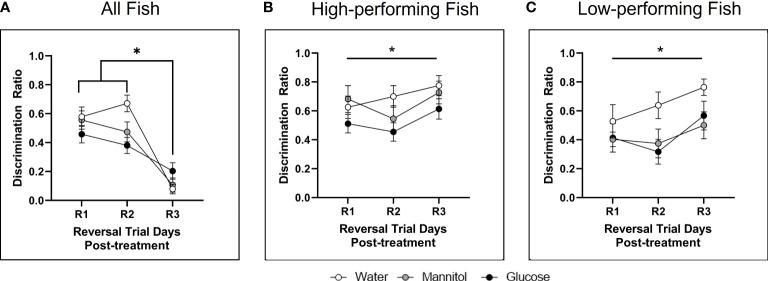
Discrimination ratio following 8-weeks of hyperglycemia. During reversal **(A)** there was a main effect of treatment (p<0.001) when all fish were analyzed together, with differences between water vs. mannitol (p=0.008) and between water vs. glucose (p<0.001). There was also a main effect of day (p=0.010) as responses during R1 and R2 were found to be different from those on R3. There was a main effect of treatment on discrimination ratio during reversal in **(B)** high performing fish (p=0.020) and **(C)** low performing fish (p=0.010). For high performers, a significant difference occurred between glucose vs. water treatment groups (p=0.019); for low performers the water treatment group significantly different from both the mannitol (p=0.024) and glucose (p=0.017) treatment groups. Data represents mean ± SE. Asterisks denote statistical significance.

There were significant main effects of both day (p=0.010) and treatment (p<0.001) on discrimination ratios of all fish combined (**Figure 6A**), with significant differences identified between R1 and R3 (p=0.022) and between R2 and R3 (p=0.003). Significant differences were also observed between water-treated controls and both the glucose and mannitol groups (water vs. mannitol: p=0.008; water vs. glucose: p<0.001); glucose and mannitol were not significantly different (p=0.265).

An effect of treatment was also observed in high performing fish (p=0.020; **Figure 6B**) due to a significant difference between glucose vs. water treatment groups (p=0.019). For low performers (**Figure 6C**), there was also a main effect of treatment (p=0.010) with the water treatment group significantly different from both the mannitol (p=0.024) and glucose (p=0.017) treatment groups. However, there were no individual differences in treatment groups for any behavioral session (R1, R2, or R3).

#### 3.2.4 Optomotor responses

As observed at the 4-week time point, OMR performance ratio of glucose-treated fish was higher than the performance ratio of mannitol-treated fish after 8-weeks of treatment. However, the 1.8x increase in performance ratio was not significantly different from controls (p=0.301; **Supplemental Figure 2B**).

#### 3.2.5 Neurochemistry

**Figure 7:**
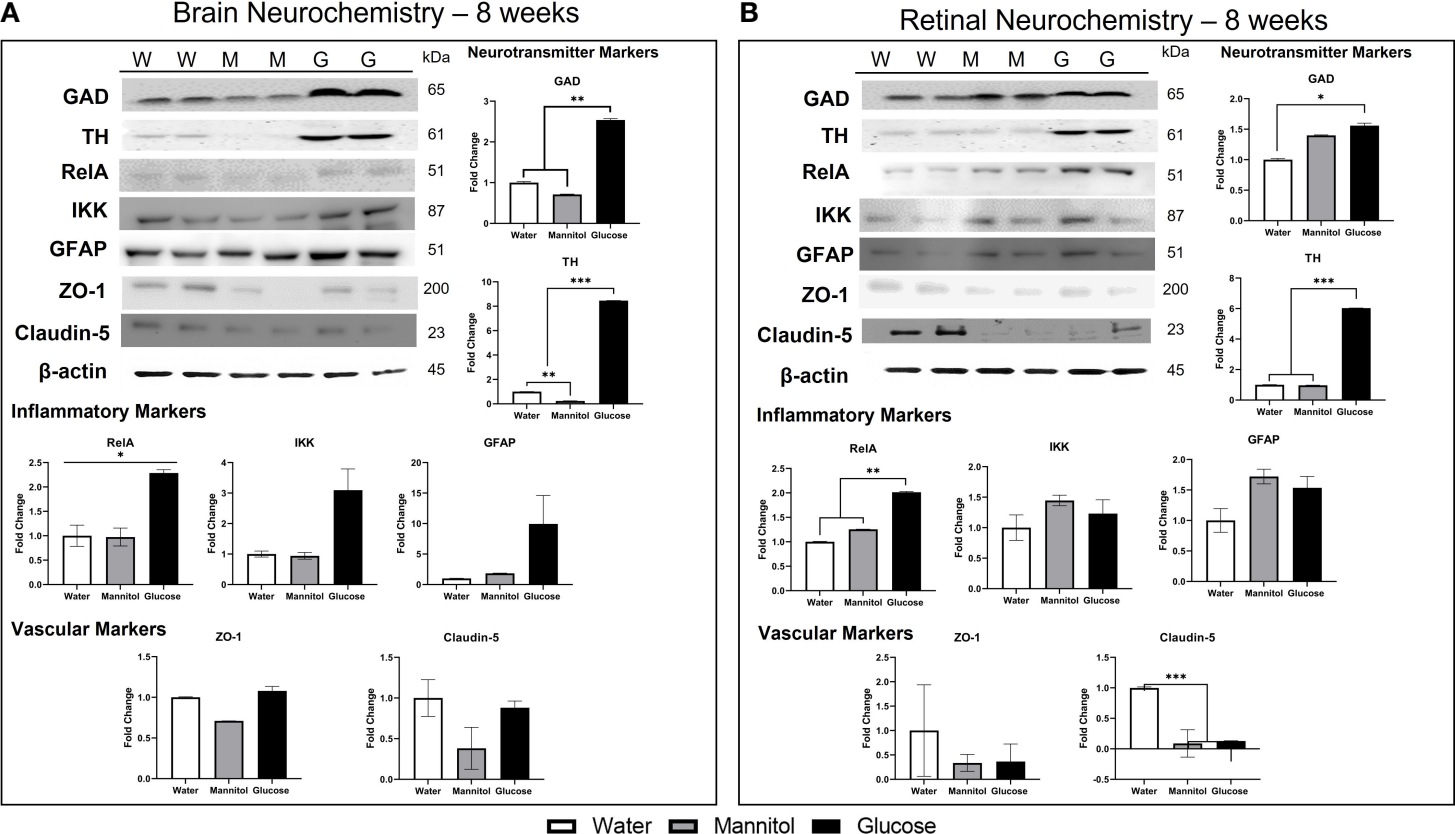
Brain and retinal neurochemistry after 8-weeks of hyperglycemia. **(A)** Brain neurochemistry after 8-weeks of hyperglycemia. Neurotransmitter markers: Both GAD and TH levels were significantly increased at this timepoint. Inflammatory markers: Levels of inflammatory markers were also increased: RelA (2.25x), IKK (3x), and GFAP (10x). Vascular markers: ZO-1 levels and claudin-5 levels were not different across treatment. **(B)** Retinal neurochemistry after 8-weeks of hyperglycemia. Neurotransmitter markers: TH levels increased 6.x in glucose-treated tissue; GAD levels were increased in both glucose- and mannitol-treated tissue. Inflammatory markers: RelA levels increased, but there was no change in IKK or GFAP levels. Vascular markers: Osmotic decreases in ZO-1 and claudin-5 were present; with claudin-5 levels in mannitol- and glucose-treated tissue significantly reduced. Representative Western Blots are shown at the top left of both panels **(A)** and **(B)**, with W, water treated; M, mannitol treated; G, glucose treated. Each sample was run in technical replicates and protein levels were normalized to β-actin. Data in all graphs are mean ± SE. Asterisks denote statistical significance. White bars, water-treated; gray bars, mannitol-treated; Black bars, glucose-treated.

In brain homogenates (**Figure 7A**), there was a significant glucose-specific increase in GAD (2.5x; p=0.011) and TH (8.5x; p<0.001) protein levels, indicating a strong effect of glucose. All inflammatory markers were also upregulated in glucose-treated tissue. RelA significantly increased by 2x (p=0.049). IKK levels increased by 4x (p=0.224) and GFAP levels increased by 10x (p=0.420-value). ZO-1 protein levels were not different among brain homogenates (p=0.065), and no difference was observed in claudin-5 levels (p=0.274). However, for both tight junction proteins, levels in glucose-treated tissue were greater than mannitol controls.

In retinal homogenates (**Figure 7B**), glucose induced a significant increase in both TH (6x; p<0.0001) and GAD (p=0.022) levels. RelA was the only inflammatory marker upregulated in retina tissue (2x; p=0.004); protein levels of IKK (p=0.796) and GFAP (p=0.665) appeared to increase osmotically and were not different across treatment groups. Claudin-5 protein was significantly reduced in both mannitol- and glucose-treated samples (p=0.001); ZO-1 protein level was also reduced in mannitol- and glucose-treated tissue, however, this difference was not significant (p=0.212).

### 3.3 *In vivo* and *in vitro* assessment of vascular changes

**Figure 8:**
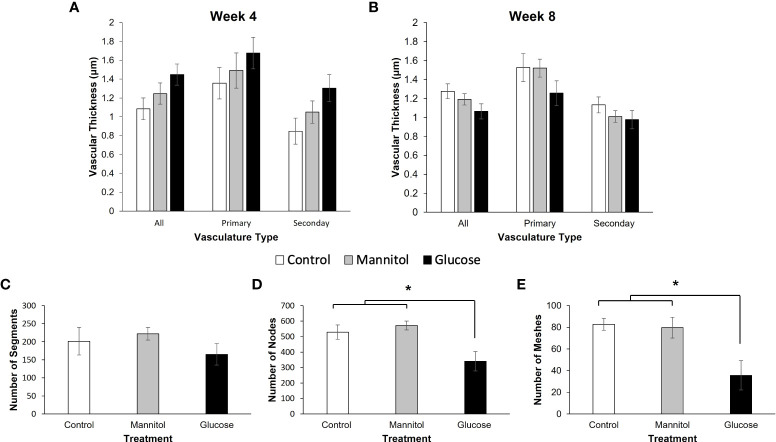
*In vivo* and *in vitro* assessment of vascular changes. Vascular thickness measured in ZO-1 labeled retinal flatmounts from fish exposed to water, mannitol, or glucose for **(A)** 4-weeks or **(B)** 8-weeks. Measurements were collected ~ 150 μm from the center of the optic nerve. After treatment for 4-weeks **(A)** there was no difference in vasculature thickness when calculated as all measurements (p=0.075), primary vessels (p=0.429) or secondary vessels alone (p=0.061). However, in all cases, vessels were thickest in the retinas of glucose-treated fish. **(B)** In contrast, after 8-weeks of exposure, glucose treatment group had the smallest vessel thicknesses, though measurements were not significantly different (all: p=0.144; primary: p=0.315; secondary: p=0.354). **(C–E)** *In vitro* tube formation assay and angiogenesis analysis of segments, nodes and meshes. **(C)** There was no difference in the number of vessel segments formed between treatment groups (p=0.441). However, there was a significantly lower number of **(D)** nodes (p=0.031) and **(E)** meshes (p = 0.002) in 3B-11 endothelial cells maintained in high glucose media. Data in all graphs are mean ± SE. Asterisks denote statistical significance.

The number of primary and secondary vascular branches across all treatment groups were comparable (**Figure 8**, **Supplemental Figure 4A**). On average, 150 μm from the center of the optic nerve there were approximately 30 branches, with about ~57% being secondary branches. After treatment for 4-weeks (**Figure 8A**), no statistically significant differences in vascular thickness were observed (all vessels combined: p=0.075; primary vessels only: p=0.429; secondary vessels only: p=0.061). However, for all measurements, the glucose treatment group exhibited the largest vascular thickness.

The number of primary and secondary vascular branches were also comparable across each treatment group at the 8-week time point (**Figure 8B**; **Supplemental Figure 4A**). On average, 150 μm from the center of the optic nerve there were 35 branches, with about 65% being secondary branches. However, at this time point, vessels in retinas from the glucose-treated fish were consistently the thinnest, though measurements were not found to be statistically significant (all: p=0.144; primary: p=0.315; secondary: p=0.354).

Within the *in vitro* tube formation assay, glucose exposure did not significantly alter the number of segments that formed (p=0.441; **Figure 8C**). However, there was a significant effect of glucose treatment on the number of nodes (**Figure 8D**; p = 0.031) and meshes (**Figure 8E**; p = 0.002). These results indicate that 3B-11 endothelial cells in the glucose treatment group failed to create a complex vascular network compared to those maintained in either control group (**Supplemental Figure 4B**).

## 4 Discussion

**Figure 9:**
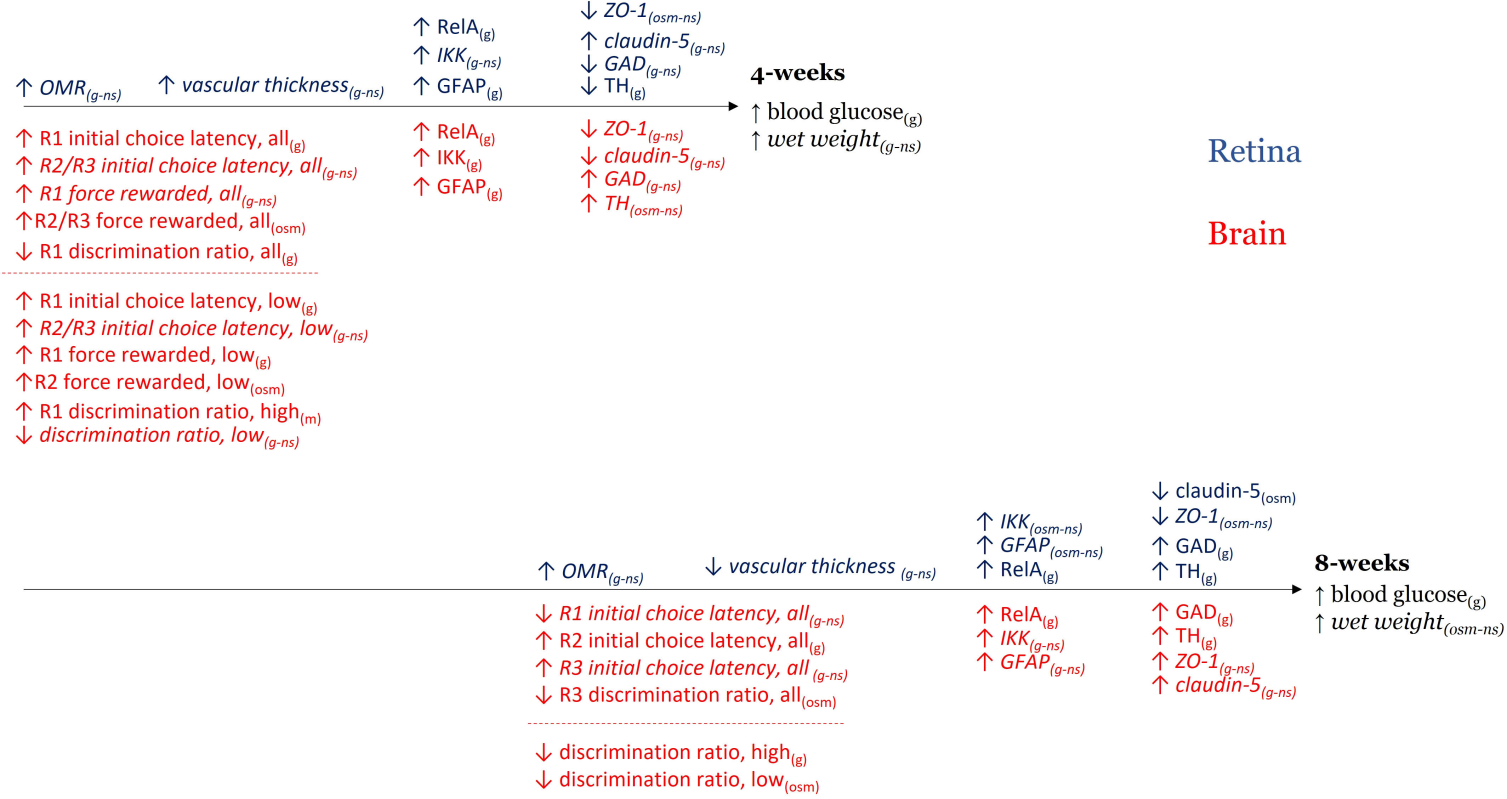
Summary of time- and tissue-dependent changes. Summary figure of hyperglycemia-induced changes in retinal/visual responses (blue) and brain/cognitive responses (red) after 4-weeks (top) and 8-weeks (bottom) of treatment. Arrows reflect direction of the difference observed. Text in parentheses denotes if the result occurred in glucose-treated tissue only (g), if the result was observed in both glucose- and mannitol treated tissue (osm – osmotic effect), or if the outcome was not significantly different across treatments (*ns, italics*). In the case of a non-significant outcome, g and osm are included to clarify trends. All = all fish; low = low performers; high = high performers.

The purpose of this study was twofold. First, we asked if there are comparable neurochemical changes in retinal and brain tissue in response to prolonged hyperglycemia in the zebrafish model and whether these changes correlated with vision- and cognition-based behaviors. Second, we assessed if continued hyperglycemic insult resulted in a worsening of effects. Overall, we observed time- and tissue-dependent changes (summarized in **Figure 9**). In both tissues, a strong initial inflammatory response was associated with non-significant decreases in ZO-1 and differential changes in claudin-5 (increased in retina; decreased in brain). Neuronal markers were initially increased in brain but decreased in retina (significantly for TH). Increases in GAD and TH levels were significant after the longer, 8-week exposure. High blood sugar also differentially impacted measured behaviors at both time points. Surprisingly, a longer duration of hyperglycemia did not always exacerbate initial effects suggesting two intriguing possibilities: 1) the fish are adapting to/compensating for the high blood sugar levels or 2) the initial and maintained inflammation has compromised tissue revealing a secondary osmotic effect.

### 4.1 Hyperglycemic induction

Consistent with previous findings (from our lab and others), at both the 4- and 8-week timepoints, blood glucose levels in the glucose treated fish were elevated 3-fold compared to either control group (18, 20). We did not, however, observe a difference in weight across groups. Uncontrolled hyperglycemia in diabetic humans and/or other animal models is known to alter the gut microbiome (41–44), reduce gut motility due to apoptotic loss of enteric neurons and decreased PI3K signaling (45), and/or induce histopathological changes in the liver (46, 47). Hyperphagia is also observed in hyperglycemic rats (48), due to altered glucose metabolism. Though not significant, the trends in measured wet weights identified here revealed increased weight in hyperglycemic fish at both the 4- and 8-week timepoint. This suggests that glucose-treated fish may have been consuming more food than fish in the other treatment groups. Though these results are in agreement with weight differences in diet-induced obesity (DIO) zebrafish (49), another Type 2 model, they do contrast previous work from our lab showing zebrafish aged 5-6 months lost a significant amount of weight after two months of hyperglycemia (19).

### 4.2 Effect on inflammatory proteins

Diabetes is associated with glucose-induced upregulation of pro-inflammatory and pro-apoptotic markers (50–53), including RelA (NF-kB) and IKK. RelA is a transcription factor and primary regulator of inflammatory responses, and IKK is necessary for the activation of RelA during acute and onset of inflammation (54). We previously reported a strong upregulation of RelA and GFAP in retina after 4-weeks of hyperglycemia (20). Here, we found the same increase in retinal GFAP, RelA, and IKK levels, though only RelA was significantly elevated in retinal homogenates after 8-weeks. A strong, consistent inflammatory response in zebrafish brain tissue was also evident after both 4- and 8-weeks of treatment. Postmortem analysis of retinas from diabetic humans (55–57) and streptozotocin-induced rats (58–64) show increased immunoreactivity patterns and/or expression of the glial marker GFAP in retinal Müller cells. Upregulation of GFAP occurs in response to hyperglycemia (56, 59, 62, 65); however, this is not an immediate response, but one that increases with time and duration of diabetes (59, 62–64). GFAP expression transiently increases in streptozotocin-induced mice (66) and mice fed a high fat and fructose diet after 4- and 24-weeks (17), in agreement with our data showing an initial significant increase in GFAP in both retina and brain. However, our findings at 4 weeks contrast a previous report which identified an increase in GFAP levels only in mannitol-treated zebrafish retinal tissue after 4-weeks of exposure (21). At our 8-week time point, the increase in GFAP levels is no longer significant in brain or retina, despite continued hyperglycemia.

Thus, we observed a strong initial inflammatory response (at 4-weeks) in both retina and brain. Inflammation was also observed after 8-weeks of hyperglycemia, though the effects were more moderate, as only Nf-κB levels remained significantly elevated in both tissues.

### 4.3 Effect on vascular tight junction proteins

The relationship between diabetes and vascular disease is long-established (67, 68). We observed a non-significant decreased ZO-1 levels in both retina and brain tissue at the 4-week time point, when claudin-5 levels were also reduced in brain. These reductions in both ZO-1 and claudin-5 levels may suggest a compromised BBB. In contrast, claudin-5 protein levels in retina appeared increased in hyperglycemic tissue after 4-weeks of treatment. Differential effects on BRB and BBB permeability were similarly noted in a Type 2 rodent model (8).

These tissue-specific differences in tight junction protein levels (increase/decrease) may be due to their specific location and function. Claudin-5 is an intermembrane protein (69) that is present in the earliest stages of central nervous system angiogenesis (70). ZO-1 is an intracellular protein responsible for the stability of tight junctions and binds other tight junction proteins, such as occludin and claudin-5, to the cytoarchitecture (71). Published reports indicate hyperglycemic zebrafish treated with 111 mM (2%) glucose for two weeks did not show a significant change in claudin-5, ZO-1a, and ZO-1b in brain homogenates (72). However, mice fed a high fat and fructose diet for 24-weeks showed significantly increased BBB permeability, heightened inflammation, and leukocyte recruitment (17). Namely, there was a downregulation of occludin-1 after 4- and 24-weeks of the high fat and fructose diet in addition to a downregulation of ZO-1 after 24-weeks. Sixty days following streptozotocin induction in rats, levels of BBB specific proteins, including occludin, claudin-5, and aquaporin-4, were significantly decreased when compared to controls, though ZO-1 was not affected (73). These reports, combined with our results showing non-significant trends after 4-weeks, suggest there is a specific time course to the loss of tight junction proteins in response to hyperglycemia.

At our 8-week time point, levels of both ZO-1 and claudin-5 in brain were highest in hyperglycemic tissue, almost doubling compared to levels at the 4-week time point. In contrast, ZO-1 and claudin-5 levels were reduced in glucose- and mannitol-treated retinas. This latter result suggests the BRB may be altered and sensitive to osmotic differences. While high glucose levels clearly lead to pathological effects, high mannitol levels do not. Mannitol is a 6-carbon sugar, like glucose, but with 2 extra hydrogen atoms (74). Clinically, mannitol is administered intravenously to reduce intraocular pressure in acute glaucoma (75–79) and as an adjuvant to facilitate drug delivery to the brain because it is able to transiently increase BBB and BRB permeability (80, 81). High extracellular glucose levels would potentially cause a similar osmotic load on endothelial cells, increasing permeability. Decreases in ZO-1 protein levels in retina at both time points were similar in glucose- and mannitol-treated tissues, suggesting an osmotic effect. Osmotic changes in claudin-5 levels, however, were only observed at the later 8-week time point. Osmotic differences also increased retinal IKK and GFAP levels after 8-weeks. A delayed osmotic effect of high glucose was unexpected and could be due to either 1) slowly developing secondary osmotic changes or 2) another mechanism that has compromised retinal tissue to such an extent that it is susceptible to osmotic differences. Our results suggest it is the second mechanism, i.e., a general osmotic changes occurring in hyperglycemic tissue after a strong initial inflammatory response.

### 4.4 Vascular morphology

Alvarez et al. (2010) reported thicker basement membranes after 28 days of hyperglycemia in adult zebrafish retina and increased vascular diameter was observed in zebrafish exposed to high glucose from 3 – 96 hpf (82). Here, we examined thickness of retinal vessels after both 4-weeks (28 day) and 8-weeks of exposure. We also used the tube formation assay to assess the ability of glucose-treated 3B-11 endothelial cells to form stable vascular networks (40).

We hypothesized that prolonged hyperglycemia would increase vascular thickness and decrease the number of vessels in the retina (83). Though we found no statistical differences in the number of vessels at either age or in vessel thickness, there was the clear trend of thicker vessels after 4-weeks of hyperglycemia, in agreement with previous reports (21). After 8-weeks, the trend reversed, with the thinnest retinal vessels observed in hyperglycemic fish. We also observed significantly less nodes and meshes in glucose-treated endothelial cell cultures, indicating the cells were unable to form a complex vascular network. Together these results are consistent with reported hyperglycemia-induced changes in retinal vasculature. In diabetic retinas, hyperglycemia causes pericyte loss and capillary dropout, triggering VEGF release and the formation of new, fragile vessels. The thinner retinal vessels observed *in vivo* may suggest a loss of larger vessels and/or the formation of smaller, new vessels. *In vitro* primary vessels were observed to form in high glucose, but they did not branch and form networks as extensively as controls. Additional experiments could examine VEGF expression in hyperglycemic zebrafish retinas at later time points to uncover vascular changes.

### 4.5 Effects on neuronal proteins

Differential changes in GAD and TH levels suggest early sensitivity differences of these transmitter systems and tissues to hyperglycemia, with levels of both proteins displaying significantly increased levels after longer exposure.

We previously showed that ERG b-waves decreased in glucose-treated tissue after 4-weeks of hyperglycemia, particularly in response to long wavelength stimuli (84). This physiological difference correlated well with red cone dystrophy reported after 28 days of hyperglycemia (21). To determine if there are additional neuronal complications in retina due to prolonged complications, we examined GAD and TH protein levels.

In hyperglycemic retina, as Müller cells become compromised, transport and/or metabolism of glutamate (85, 86), GABA (26, 27, 87), and other extracellular components are altered, causing an associated change in overall concentration and immunoreactivity patterns (26, 27, 30). Published studies report differential effects of glucose insult on both GABAergic and dopaminergic systems. Increased GABA levels are observed in vitreal samples from individuals with progressive diabetic retinopathy (58, 88) and in streptozotocin-induced rats (26). Other studies, also in rats, report an overall decrease in GABA content (89, 90). Increased GABA concentration and/or immunoreactivity patterns (26, 27, 29, 30) are associated with altered GABA-evoked responses and GABA_C_ receptor subunit expression in retinal bipolar cells (28, 29), indicating that inhibitory GABAergic transmission is altered in hyperglycemic conditions. Retinal dopamine levels (31, 32), light–evoked dopamine release (32), and diurnal dopamine rhythms (31) are decreased in other animal models of diabetic retinopathy. A similar reduction in the activity and/or immunoreactivity patterns of TH (91–93) may underlie the reported changes in dopamine levels. However, other studies identify no glucose-induced change in TH activity (32, 94) suggesting the change in dopamine concentration is due to a reduction in the concentration of precursor molecules required for dopamine synthesis (94, 95) rather than a change in enzyme activity.

Similar to other vertebrates, in zebrafish retina, GAD is expressed in horizontal and amacrine cells (96, 97); TH is found in interplexiform (97) and some amacrine cells (84). GABA and dopamine release from these cell types, respectively, modifies bipolar cell responses and subsequent input to ganglion cells. We observed decreases in retinal TH and GAD levels at 4-weeks, but significantly increased levels in retinas from hyperglycemic fish at 8-weeks. This implies an initial decrease in GABAergic and dopaminergic signaling that is followed by later enhancement, possibly reflecting a compensatory response at the later age. Surprisingly, the lower levels of GAD and TH at 4-weeks, does not correlate with the observed decrease in b-wave amplitude (84) suggesting more than one effect of hyperglycemia in retina.

To further assess neuronal changes, we probed blots of 4-week retinal homogenates with an antibody to PKCα, a standard marker for ON-bipolar cells. Our logic was that since ON-bipolar cells generate ERG b-waves, the reduced b-wave amplitudes reported at 4-weeks may be correlated with a reduction in PKCα levels. However, PKCα levels were increased in retinas collected from both mannitol- and glucose-treated fish (**Supplemental Figure 5**). This result could reflect (1) the presence of PKCα in other retinal cell types and/or (2) a generalized upregulation of PKCα in response to treatment. PKCα antibodies do label ON-bipolar cell types in zebrafish, including both cone-only and mixed input cells (98, 99), as well as rod bipolar cells in mammals (100–102). However, amacrine cell, ganglion cell, and/or photoreceptor labeling has been also reported (98, 100–102), suggesting the increased levels we observed may reflect changes is these other cell types. PKC levels in retina vessels are very sensitive to hyperglycemia (103, 104), with activated/increased PKC associated with decreased blood flow and increased permeability (104). Though a different isoform, PKCβ, seems to be preferentially activated by high glucose, PKCα levels do increase in retina (104). Thus, the increased PKCα levels we observed could reflect an overall retinal response to osmotic load, and not one associated with a specific cell type. Additional experiments are needed to differentiate between PKCα effects in hyperglycemic zebrafish retinas

In brain homogenates, the early, and later significant, increase in both GAD and TH protein levels suggest a worsening of effects over time. In diabetic patients, increased concentrations of GAD antibodies are detected in serum samples and associated with the loss of pancreatic beta cells (105) and development of insulin dependence (106). Increased levels of GAD also suggest increased synthesis of GABA. In fact, GABA administration before symptom onset is protective promoting beta cell replication and survival in both STZ-induced diabetic and NOD mice (107). Further, GABA administration after symptom onset reversed disease progression and reduced inflammation (107). The increased GAD levels we observed after 8-weeks of hyperglycemia occurred when increases in IKK and GFAP protein levels were no longer significant, possibly due to anti-inflammatory effects of increased GABA levels in brain.

High intake and levels of glucose (carbohydrates) stimulate dopamine release, with significant correlations between blood glucose levels and dopamine metabolites in spinal fluid (108). Experimental rats made hyperglycemic by daily glucose injections for 30 days show increased dopamine concentration in the striatum and hippocampus (109). However, prolonged hyperglycemia (6 months after STZ injection) can reduce brain dopamine levels due to the loss of dopaminergic neurons (110). In this situation, high TH levels are a compensatory response to the loss of dopaminergic neurons (111) caused by continued inflammation. Our behavioral data at the 8-week time point does not suggest neuronal loss, though TH levels were strongly increased. A longer duration of hyperglycemia and/or different behavioral assessments may be needed to see if dopaminergic neurons are lost in hyperglycemic zebrafish brains.

### 4.6 Effects on behavioral measures

The optomotor response is a vision-based behavior used to test whether there are deficits in the retinotectal pathway in the zebrafish (112, 113). Given that hyperglycemic zebrafish have functional deficits in the retina (20, 21), we hypothesized that there may also be deficits in visually-guided behaviors that involve downstream pathways. Our results indicate that glucose-treated fish have a better OMR than controls at both time points. This result was surprising given the neurochemical changes and may occur because the circuitry for the OMR is not exclusively retina-based (112) and/or that the OMR is not sensitive enough to detect hyperglycemia-induced changes in higher order visual circuits. Importantly, the robust OMR in the glucose-treated group can also serve as an effective positive control for the three-chamber choice task which used a visual cue to direct the fish to the proper chamber.

While all fish responded to the OMR, during the cognitive assessment we noticed that some fish were able to learn the task more quickly and accurately than others. The responses of these ‘high performing fish’ were analyzed separately due to the large variability observed when all responses were pooled together. Zebrafish have been similarly classified in studies examining effects of alcohol exposure, revealing that ‘bold’ vs. ‘shy’ fish (114) and fish that hatch early vs. fish that hatch later (115) have different susceptibilities to alcohol. Our results suggest low and high performing zebrafish show differential susceptibility to hyperglycemic insult. Glucose-treated low performing fish displayed behavioral responses different from either control group after 4-weeks of exposure. However, response differences in high performing fish were not observed until after 8-weeks of treatment. These results suggest a time-dependent component to glucose-induced effects and, potentially, differential susceptibility between fish classified by their performance.

Further, at the 4-week time point, glucose-treated fish took longer to make their initial decision and the increased number of force-rewarded trials suggests that some glucose-treated fish were unable to decide at all. Significant differences in the response of low performing fish were particularly evident after treatment, when choice latency was increased on R1, and the number of force-rewarded fish was increased on all reversal days. Discrimination ratios were also lower for glucose-treated fish, indicating they were unable to reverse their learning of the task. The effect on initial choice latency is observed in only the glucose-treated group, while the increase in force-rewarded fish was observed in both mannitol- and glucose-treated groups. In contrast, fewer significant differences were observed overall after 8-weeks of treatment. Most differences identified at this time reflected differences with the water-treated group; responses of glucose- and mannitol-treated groups were comparable.

Thus, significant behavioral differences between the glucose and control groups were evident after 4-weeks of exposure and are primarily seen in low performing fish. At the 8-week time point, most responses of glucose-treated fish were comparable the mannitol-treated controls, suggesting either compensation or a secondary osmotic response.

### 4.7 Does hyperglycemia cause similar effects in zebrafish brain and retina?

Combining the above information indicates hyperglycemia does impact the same neurochemical markers in both zebrafish brain and retina. However, the noted changes in brain and/or retina depend on duration of exposure, and which tissue is assessed. Opposite trends were noted for neurotransmitter markers in brain and retina at 4-weeks. Further, it was not always possible to correlate significant neurochemical changes with significant behavioral deficits (**Figure 9**).

For example, after 4-weeks, we observed a strong glucose-specific increase in RelA, IKK, and GFAP in brain tissue. Protein levels of ZO-1 and claudin-5 were decreased, while levels of GAD and TH were elevated. These indicate inflammation and suggest an initial increase in vascular permeability and some altered neuronal activity. After 8-weeks, brain tissue displayed a more moderate glucose-induced increase in all 3 inflammatory markers while GAD and TH levels were now significantly elevated, suggesting continued/increased damage. ZO-1 and Claudin-5 levels remained statistically similar, though levels were now increased. These data suggest that in zebrafish brain tissue 1) hyperglycemia induces and maintains an inflammatory response, 2) glucose-driven damage to GABAergic and dopaminergic neurons increases with time, and 3) tight junction proteins are differentially affected by high glucose.

In retina, a strong inflammatory response after 4-weeks of hyperglycemia was also observed. This response was accompanied by a significant decrease in TH protein levels. Tight junction proteins were differentially affected, with a non-significant increase in claudin-5, and an osmotic reduction in ZO-1. After 8-weeks, though RelA levels were still increased, protein levels of the other inflammatory markers were not different from controls and osmotic differences were observed for ZO-1 (non-significant decrease), claudin-5 (significant decrease), and GAD (significant increase). Interestingly, there was a strong glucose-induced increase in retinal TH levels at this time, suggesting retinal dopaminergic cells may be uniquely sensitive to prolonged glucose insult. Thus, in retinal tissue 1) hyperglycemia induces an initial inflammatory response that is reduced with prolonged exposure, 2) dopaminergic circuitry is primarily affected, and 3) tight junction proteins are decreased in both glucose- and mannitol-treated tissue suggesting an osmotic effect.

Behaviorally, glucose-treated fish displayed an increased OMR, took longer to make a first decision in the 3-chamber choice task, and had reduced discrimination ratios indicating reduced cognitive performance after 4-weeks. After 8-weeks of hyperglycemia, OMR performance ratio and increased choice latency remained increased in glucose-treated fish whereas, osmotic differences decreased discrimination ratios.

These results suggest that 4-weeks of hyperglycemia causes changes in zebrafish retinal and brain tissue that are similar to effects reported in other animal models. These differences were correlated with decreased cognitive responses. Surprisingly, however, a longer duration of hyperglycemia seemed to mitigate many of these effects. The strong increase in GAD and TH levels in both retina and brain at 8-weeks, coupled with elevated high blood sugar levels and maintained inflammation, suggests a continued pathology. However, the other parameters displaying significance at 4-weeks were either no longer significant across treatments or displayed secondary osmotic effects.

Overall, there appear to be time- and tissue-specific effects of prolonged glucose exposure in hyperglycemic zebrafish. Our results suggest that inflammation is an early cellular response to increased blood sugar levels. Continued glucose exposure maintains the inflammatory response and leads to significant changes in neurochemical markers. Osmotically driven changes affect tight junction protein levels in retina to a greater extent than in brain. For some markers assessed, initial glucose-specific differences observed at 4-weeks became osmotically driven at 8-weeks. This suggests either the development of secondary osmotic effects with continued hyperglycemic insult or that the fish may be compensating for glucose exposure. Current experiments are underway to address these two hypotheses.

## Data availability statement

The raw data supporting the conclusions of this article will be made available by the authors, without undue reservation.

## Ethics statement

The animal study was reviewed and approved by Institutional Animal Care and Use Committee (IACUC) at American University (protocol #1606, #1902).

## Author contributions

CR: Conceptualization; Data curation; Formal analysis; Methodology; Project administration; Visualization; Writing - original draft; Writing - review & editing. MD-P, NB, and AC: Methodology. MB and KD-S: Conceptualization; Methodology; Supervision. TD: Conceptualization; Writing - review & editing. VC: Conceptualization; Visualization; Writing - original draft; Supervision; Writing - review & editing; Funding acquisition. All authors contributed to the article and approved the submitted version.

## Funding

This work was supported by a Faculty Research Support Grant to VC; CR received funding from the American University College of Arts and Science Graduate Student Research Support for research supplies and the American University Center for Behavioral Neuroscience Summer Research Award.

## Acknowledgments

The authors thank Sabrina Jones for her assistance adapting a rodent three-chamber choice paradigm to the zebrafish model and Allison Murk and Jeremy Popowitz for help on behavior collection days, assistance with running trials, animal care, and tank set-up. Special thanks also to James M. Forbes (Engineer) for his assistance with the 3-chamber choice tank design and construction. This article was written by CR in partial fulfillment of the requirements in pursuit of the degree of Doctor of Philosophy in Behavior, Cognition, and Neuroscience at The American University.

## Conflict of interest

The authors declare that the research was conducted in the absence of any commercial or financial relationships that could be construed as a potential conflict of interest.

## Publisher’s note

All claims expressed in this article are solely those of the authors and do not necessarily represent those of their affiliated organizations, or those of the publisher, the editors and the reviewers. Any product that may be evaluated in this article, or claim that may be made by its manufacturer, is not guaranteed or endorsed by the publisher.

## Supplementary material

Supplementary Table 1 Neurochemistry and Immunohistochemistry Antibodies. Primary and secondary antibodies used in this study for Western blots and immunohistochemistry.

Supplementary Figure 1 Initial choice latency and force-rewarded trials of high vs. low performing fish after 4-weeks of glucose treatment. **(A)** High performing fish. There were no differences in initial choice latency during contrast discrimination reversal (p=0.879; top panel). The number of force-rewarded trials was also not significantly different (bottom). **(B)** Low performing fish. There was a significant difference in initial choice latency during reversal (R1, p=0.002, top panel), with glucose-treated fish taking significantly longer to make an initial decision compared to controls. There was a main effect of treatment for the number of force rewarded trials during reversal on all three days (p<0.001; bottom right). Data is represented as averages per fish per training day ± SE. Asterisks denote statistical significance.

Supplementary Figure 2 Optomotor responses after 4-weeks and 8-weeks of treatment. OMRs were evoked in treated zebrafish as well as from fish directly removed from stock (AHAB) tanks. We added stock fish as an additional handling control. Performance ratio for each treatment group (top panels) were normalized to the stock handling control (bottom panels) prior to statistical analysis. Similar trends in OMR were observed at the 4-week **(A)** and 8-week **(B)** timepoints. There were no statistical differences across groups for either time point. Values are mean ± SE.

Supplementary Figure 3 Initial choice latency and force-rewarded trials of high vs. low performing fish after 8-weeks of glucose treatment. **(A)** High performing fish displayed no differences in initial choice latency during reversal (p=0.608, top panel). However, during reversal, high performing fish showed a main effect of treatment (p=0.036) and day (p=0.041), with more marked fish on R2 compared to R3 (bottom panel). **(B)** Low performing fish displayed no differences in any parameters: initial choice latency during reversal (p=0.608; top panel) and the number of force-rewarded trials during reversal (bottom panel). Bars represent mean values ± SE. Asterisks denote statistical significance.

Supplementary Figure 4 Micrographs of retinal flatmounts and in vitro endothelial cells to assess vascular changes. **(A)** Flat mounted retinas collected from zebrafish exposed to water, mannitol, or glucose for either 4-week or 8-week. Flatmounts were stained with ZO-1 antibody to examine the vascular network. Images were taken with Olympus BX61 at 20x magnification using the GFP filter. Images are positioned at the optic nerve and vascular network was quantified ~150 μm from the center of the optic nerve. Arrows highlight retinal vessels. **(B)** 400 x 400 dpi microscope images of tube formation by 3B-11 endothelial cells cultured in either control (unconditioned) media or media supplemented with mannitol or glucose. Cultures were imaged at the 6.5 hr timepoint. Images were used to calculate the formation of nodes, branches, and meshes within each treatment group.

Supplementary Figure 5 PKCα levels in retinal homogenates after 4-weeks of hyperglycemia. **(A)** Western Blots showing differences in PKCα protein levels across treatments. PKCα levels were significantly increased in both mannitol- and glucose-treated tissue. **(B)** Fold change in protein levels determined by densitometry. Each sample was run in technical replicates and protein levels were normalized to β-actin. Data in **(B)** is mean ± SE. Asterisk denotes statistical significance. White bars = water-treated, gray bars = mannitol-treated, Black bars = glucose-treated.
