# Supplementary material for: Time dependent effects of prolonged hyperglycemia in zebrafish brain and retina
Source: Front Ophthalmol (Lausanne). 2022 Aug 25;2:947571. doi: 10.3389/fopht.2022.947571 (PMC11182107; doi:10.3389/fopht.2022.947571)
Supplement: Supplementary Table 1 [file Table_1.docx]

|  | **Antibody** | **Catalog #** | **Vendor** | **Dilution** | **Secondary AB** |
| --- | --- | --- | --- | --- | --- |
| **Primary Antibodies** |  |  |  |  |  |
|  | β-actin | 4967S | Cell Signaling Technology | 1:2000 | Anti-Rabbit 1:2000 |
|  | Glutamate Decarboxylase (GAD) | AB1511 | EMD Milipore Sigma | 1:5000 | Anti-Mouse 1:2500 |
|  | Tyrosine Hydroxylase (TH), clone LNC1 | MAB318 | EMD Milipore Sigma | 1:1000 | Anti-Mouse 1:2000 |
|  | V-Rel Avian Reticulodneotheliosis Viral Oncogene Homolog A (RelA) | 55482 | AnaSpec | 1:500 | Anti-Rabbit 1:2000 |
|  | IkB kinase (IKK) | 55481 | AnaSpec | 1:500 | Anti-Rabbit 1:2000 |
|  | Zonula Occludin-1 (ZO-1) | 61-7300 | Thermofisher Scientific | 1:1000 | Anti-Rabbit 1:2000 (WB); Anti-Rabbit 1:200 (IHC) |
|  | Claudin-5 4C3C2 | 35-2500 | Thermofisher Scientific | 1:500 | Anti-Mouse 1:2000 |
|  | Glial fibrillary acidic protein (GFAP) | MAB360 | EMD Milipore Sigma | 1:1000 | Anti-Mouse 1:2000 |
|  | Protein Kinase C alpha (PKCα) | NB600-201 | Novus Biologicals | 1:1000 | Anti-Mouse 1:2000 |
| **Secondary Antibodies** |  |  |  |  |  |
|  | Anti-Mouse IgG HRP-linked | 7076 | Cell Signaling Technology | See above |  |
|  | Anti-rabbit IgG, HRP-linked | 7074 | Cell Signaling Technology | See above |  |
|  |  |  |  |  |  |
