# Supplementary figures and images for: Time dependent effects of prolonged hyperglycemia in zebrafish brain and retina
Source: Front Ophthalmol (Lausanne). 2022 Aug 25;2:947571. doi: 10.3389/fopht.2022.947571 (PMC11182107; doi:10.3389/fopht.2022.947571)

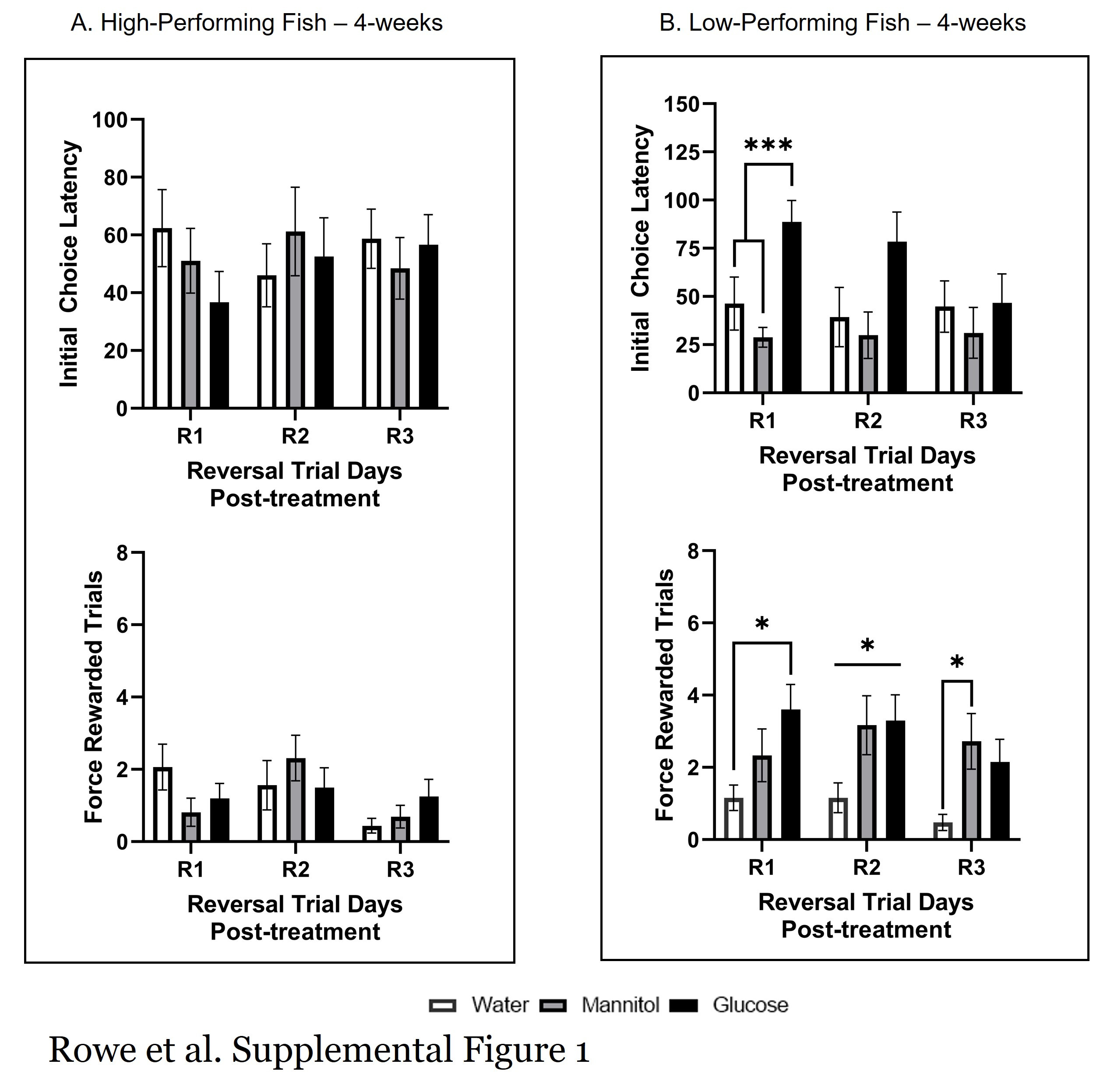

Supplement: Supplementary Figure 1 [file Image_1.jpeg]

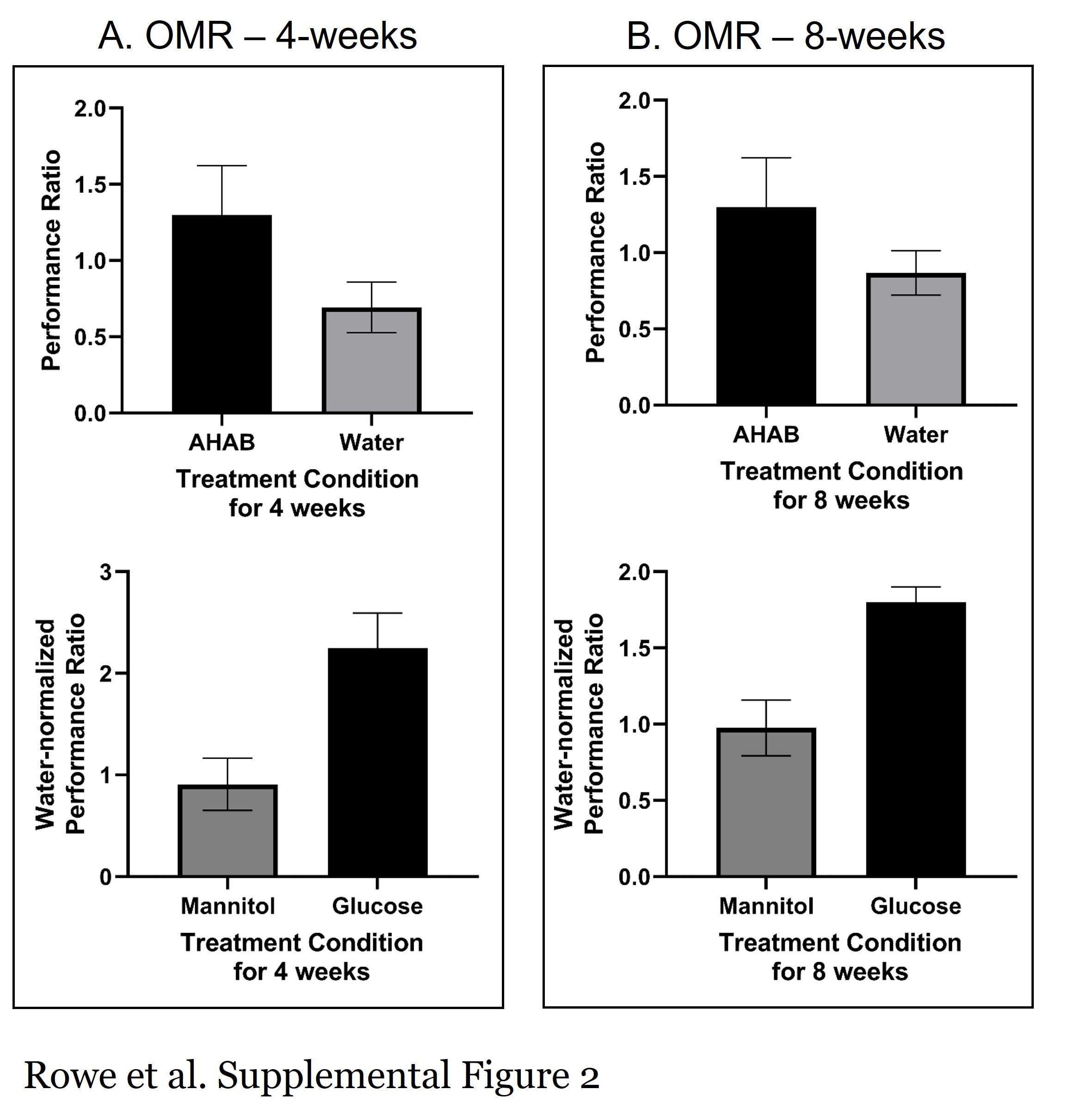

Supplement: Supplementary Figure 2 [file Image_2.jpeg]

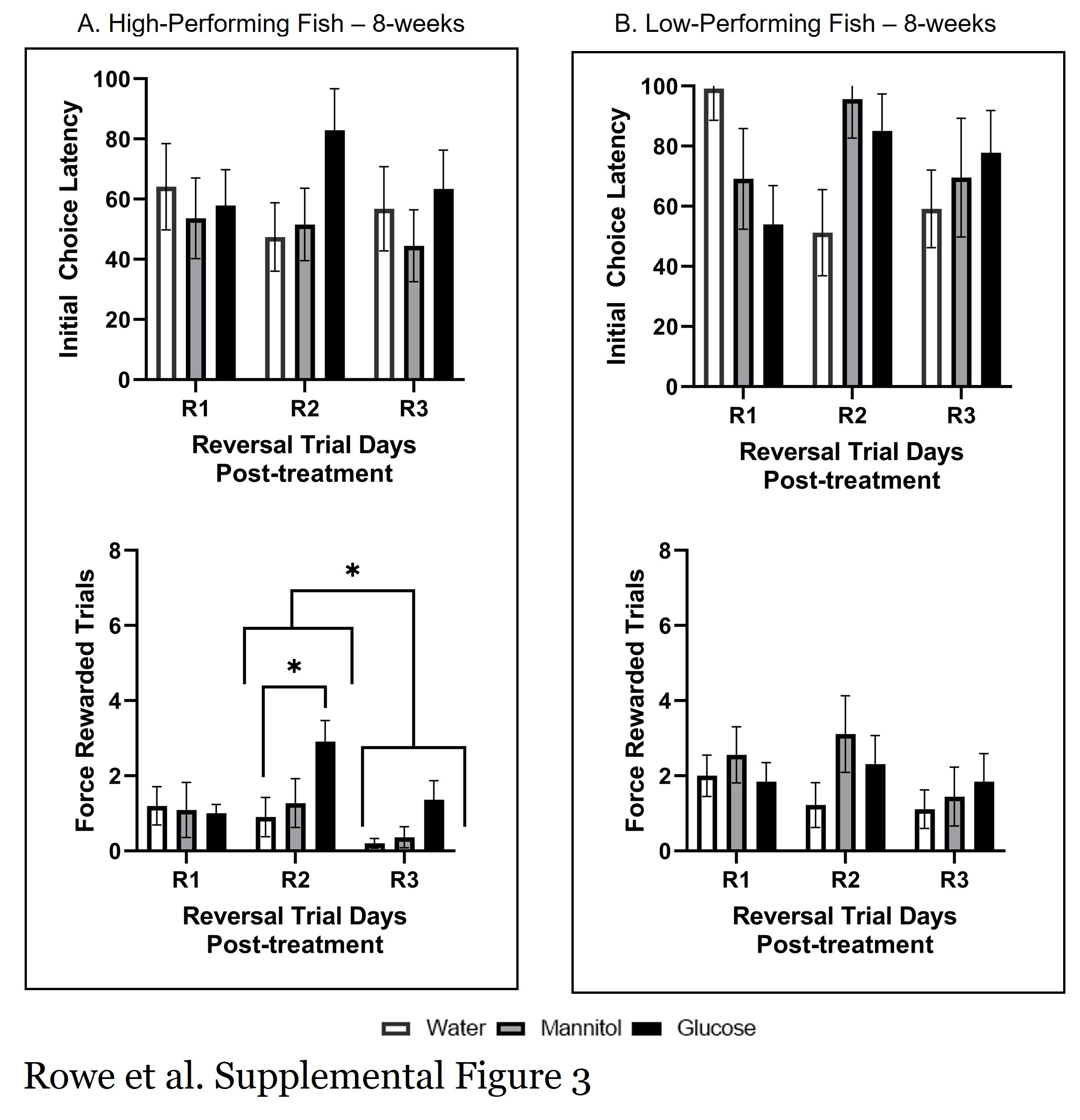

Supplement: Supplementary Figure 3 [file Image_3.jpeg]

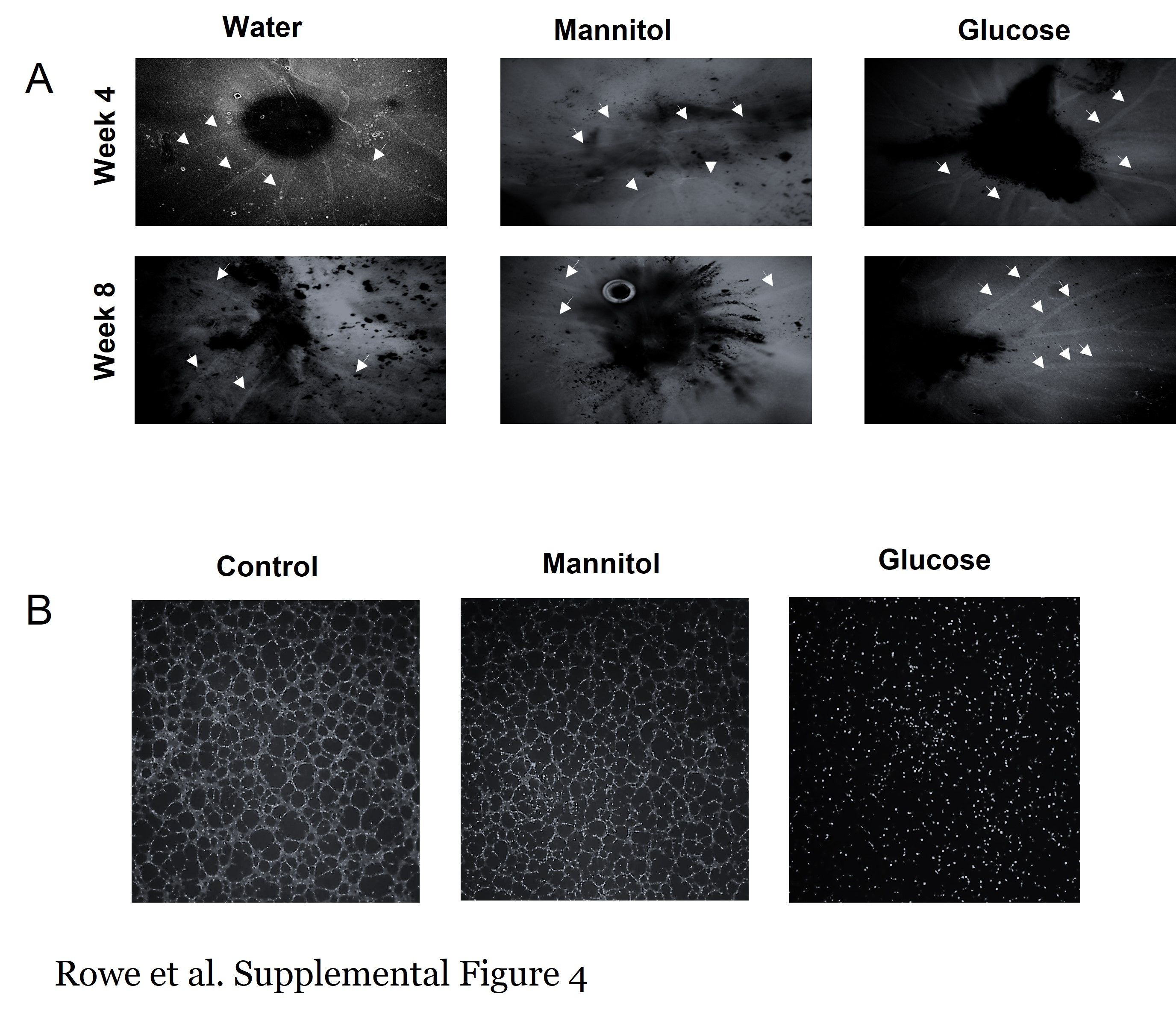

Supplement: Supplementary Figure 4 [file Image_4.jpeg]

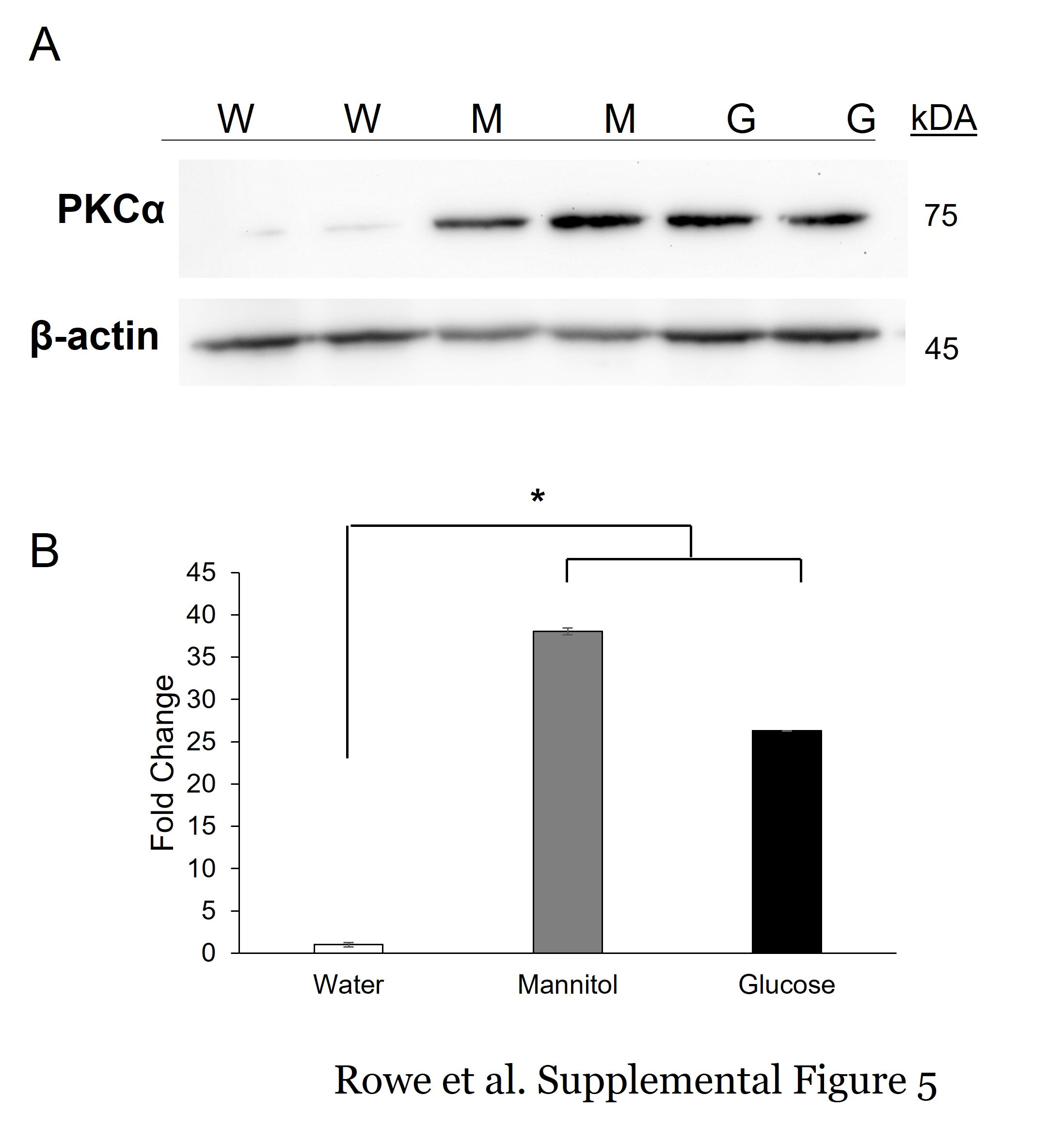

Supplement: Supplementary Figure 5 [file Image_5.jpeg]
